# Recrudescence of tuberculosis in the state of São Paulo post-COVID-19: trends and clusters

**DOI:** 10.11606/s1518-8787.2025059006987

**Published:** 2026-01-09

**Authors:** Reginaldo Bazon Vaz Tavares, Nathalia Zini, Yan Mathias Alves, André Luiz Teixeira Vinci, Natacha Martins Ribeiro, Ariela Fehr Tártaro, Valter Chicalo António Caripa, Maria Eduarda Pagano Pelodan, Clara Ferreira de Souza, Aline Aparecida Monroe, Jaqueline Garcia de Almeida Ballestero, Ione Carvalho Pinto, Pedro Fredemir Palha, Ricardo Alexandre Arcêncio

**Affiliations:** I Universidade de São Paulo. Escola de Enfermagem de Ribeirão Preto. Departamento de Enfermagem Materno-Infantil e Saúde Pública. Ribeirão Preto, SP, Brasil

**Keywords:** Tuberculosis, COVID-19, Recrudescence, Trends, Public Health

## Abstract

**OBJECTIVE:**

To analyze the recrudescence of tuberculosis in the state of São Paulo after the COVID-19 pandemic, identifying temporal trends and spatial clusters of the disease.

**METHODS:**

An ecological study of tuberculosis cases reported on TBWeb in all São Paulo municipalities between January 2015 and December 2023. Time decomposition techniques, interrupted time series analysis by month and spatial analysis by municipality (global Moran index and Getis-Ord Gi*) were applied to identify trends, abrupt changes associated with the pandemic and clusters of high incidence, mortality, and treatment outcomes. The pre-pandemic (01/2015 to 01/2020), pandemic (02/2020 to 04/2022), and post-pandemic (05/2022 to 12/2023) periods were analyzed separately.

**RESULTS:**

There was an upsurge in tuberculosis in the post-pandemic period, with a 21.2% increase in the number of municipalities with an incidence > 110 cases per 100,000 inhabitants. There was a progressive increase in the mortality trend of 0.0026 (95%CI: 0.0016 to 0.0035) deaths per 100,000 inhabitants per month after the pandemic. There was a gradual drop of 0.67% (95%CI: -1.099 to -0.246) per month in the proportion of people cured after the pandemic. High incidence clusters persisted in the Presidente Prudente region in all periods, and new clusters in Marília and Registro after the pandemic. Areas with high mortality rates persisted in the regions of Taubaté, Baixada Santista, Grande São Paulo, Registro, Sorocaba and Campinas in all periods.

**CONCLUSION:**

The recrudescence of tuberculosis in São Paulo in the post-pandemic context highlights the need for targeted strategies for early diagnosis, strengthening treatment and intensive monitoring in regions identified as clusters, especially those with vulnerable populations and structural challenges in health services.

## INTRODUCTION

Tuberculosis, although curable, remains one of the world’s public health challenges^1^. In 2024, the World Health Organization reported more than 10.8 million cases of tuberculosis and 1.25 million deaths from the disease worldwide^2^.

Brazil is one of the countries with a high burden of the disease, ranking 19th in the world in terms of number of cases, reporting 84,308 cases in 2024, which represents an incidence of 39.7 cases per 100,000 inhabitants^3^; and a 21% increase in the number of new cases in the post-COVID-19 pandemic period. Notably, the state of São Paulo accounted for approximately 25.7% of cases in Brazil in 2024, representing a critical scenario for tuberculosis control^3^.

The concept of tuberculosis recrudescence, adopted in this study, is defined as the worsening of the epidemiological scenario of the disease after the acute phase of the COVID-19 pandemic, characterized by an increase in loss to follow up, deaths, and an increase in the number of cases, including multidrug-resistant tuberculosis^4^.

The COVID-19 pandemic has posed significant challenges on health systems^5^, especially in regions with a high tuberculosis burden. Social distancing measures, the overload of services and the prioritization of pandemic control have compromised the continuity of case follow-up, particularly affecting vulnerable populations^5^.

The goals set by the “End TB*”* strategy of eliminating tuberculosis by 2050 (< 1 case per 100,000 inhabitants) have been profoundly impacted by the COVID-19 pandemic, which has imposed on the Brazilian Unified Health System (SUS), at its different levels of management (federal, state, and municipal), the implementation of strategic measures to recover the epidemiological situation and eliminate the disease, in accordance with the commitments signed at the 2nd United Nations high-level meeting on the fight against tuberculosis^6^.

There’s a need to align policies, restructure health services in synergy with social assistance and other strategic sectors in order to regain control of declining incidence and mortality, following the Sustainable Development Goals, notably item 3.3^3^.

However, many states were unable to organize themselves with regard to these guidelines and the challenges, which were already significant in the pre-pandemic period, intensified in the post-pandemic period, with risks of a resurgence of tuberculosis due to the disproportionate impacts on vulnerable populations^7^.

In the state of São Paulo, interruptions in diagnosis particularly affected peripheral communities, prison units, and homeless populations, where barriers of access to diagnosis, treatment, and follow-up aggravate underreporting^8^. These gaps possibly persisted in the post-pandemic period, with an uneven recovery of local healthcare and health surveillance systems, which could lead to a recrudescence of the disease. Further studies focusing on this issue are needed.

Previous studies have analyzed the impact of COVID-19 in the context of tuberculosis, but have not addressed the dynamics in a dual manner (time and space) for the post-pandemic^7,8^. This study therefore makes an original contribution by investigating the recrudescence of tuberculosis in the post-pandemic period.

This approach allows for the correlation of local factors, providing more comprehensive evidence of the multidimensional dynamics of the disease, as opposed to traditional analyses based on aggregated data. Thus, the aim of the study is to analyze the recrudescence of tuberculosis in the state of São Paulo in the post-COVID-19 pandemic period, temporal trends, and spatial clusters of the disease.

## METHODS

### Study Design

This is an exploratory ecological study with time series and spatial analysis^9^. The 645 municipalities in the state of São Paulo were used as the unit of analysis.

### Study Population

This study analyzed all reported cases of tuberculosis in the state of São Paulo, covering all age groups and clinical forms of the disease, from January 2015 to December 2023. To assess recrudescence, the period analyzed was divided into three distinct phases: (i) Pre-pandemic (January 2015 to January 2020); (ii) Pandemic (February 2020 to April 2022); and (iii) Post-pandemic (May 2022 to December 2023).

The start of the pandemic period was defined as February 2020, the date of the first reported case of COVID-19 in Brazil and in the state of São Paulo^10^. The end of this period was established in April 2022, when the national public health emergency related to COVID-19 was officially declared over^11^.

The tuberculosis data were obtained from the *Sistema de Controle de Pacientes com Tuberculose* (TBWeb – Tuberculosis Patient Control System), made available by the São Paulo State Health Department and the Tuberculosis Division of the “Prof. Alexandre Vranjac” Epidemiological Surveillance Center of the state of São Paulo. The population estimates used for analysis were obtained from *the Instituto Brasileiro de Geografia e Estatística* (IBGE – Brazilian Institute of Geography and Statistics)^12^.

### Study Setting

The state of São Paulo is located in the southeastern region of Brazil and is the most populous in the country, with more than 44 million inhabitants in 2022, which represents approximately 22% of the Brazilian population. Additionally, the state’s 645 municipalities are administratively organized into 63 micro-regions and 17 Regional Health Departments^12^.

### Classification of Variables

The variables used in the study were organized according to the type of analysis carried out: descriptive, temporal, and spatial.

### Variables Used for Descriptive Analysis

The variables were obtained from the Tuberculosis Notification Form of the TBWeb System, namely: (i) sex, (ii) age group in years (0–14,15–29,30–39, 40–49, 50–59 and ≥ 60 years), (iii) race/skin color (White, Brown/Mixed race, Black, Yellow, and Indigenous), (iv) education in years of schooling (no schooling, 1–3, 4–7, 8–11, 12–14, ≥ 15 years), (v) type of entry, (vi) diagnosis (rapid molecular test, histopathology, smear microscopy, culture, HIV test, drug susceptibility test, x-ray), (vii) clinical form, (viii) case outcome, (ix) antiretroviral therapy, (x) comorbidity (Aids/HIV, diabetes, drug addiction, alcoholism, smoking), (xi) homeless population and incarcerated population.

### Variables Used for Temporal Analyses

For this phase, notified cases and deaths from tuberculosis were aggregated according to the population size of the municipalities and the criteria of the São Paulo State Social Development Secretariat (SEDS-SP)^13^, namely: (a) metropolis: population of more than 900.001 inhabitants; (b) large size: population between 100,001 and 900,000 inhabitants; (c) medium size: population between 50,001 and 100,000 inhabitants; (d) small size I: population of up to 20,000 inhabitants; and (e) small size II: population from 20,001 to 50,000 inhabitants. The categorization allowed the analysis of epidemiological trends, according to the demographic profile of the municipalities.

The tuberculosis incidence rate was calculated according to the formula:


$$\frac{\text{ number of cases month }}{\text{ population }}*100,000\text{ inhabitants }$$

The tuberculosis mortality rate was calculated using the formula:


$$\frac{\text{ number of deaths month }}{\text{ population }}*100,000\text{ inhabitants }$$

In addition, it was calculated:

The proportion of tuberculosis deaths:


$$\frac{\text{ number of deaths month }}{\text{ cases of tuberculosis month }}*100$$

The proportion of tuberculosis cured:


$$\frac{\text{ number of cases cure month }}{\text{ cases of tuberculosis month }}*100$$

The proportion of tuberculosis cases lost to follow-up:


$$\frac{\text{ number of cases lost to follow-upmonth }}{\text{ cases of tuberculosis month }}*100$$

### Variables Used for Spatial Analysis (Spatial Clusters)

In order to identify critical areas of tuberculosis recrudescence and spatial patterns, tuberculosis incidence and mortality rates were estimated by municipality, as well as the proportions of cure, death, and loss to follow-up, considering four periods: (i) total study, (ii) pre-pandemic, (iii) pandemic, and (iv) post-pandemic.

Tuberculosis incidence and mortality rates were calculated according to the formulas below:

Tuberculosis incidence rate:


$$\frac{\text{ number of cases in municipality/months }}{\text{ population in municipality }}*100,000\text{ inhabitants }$$

Tuberculosis mortality rate:


$$\frac{\text{ number of deaths in municipality/months }}{\text{ population in municipality }}*100,000\text{ inhabitants }$$

### Descriptive Analysis

Sociodemographic and clinical-epidemiological variables were analyzed using descriptive statistics for quantitative parameters, and absolute and relative frequencies were calculated for the variables.

Descriptive analyses were carried out using the R^®^ language (version 4.0.3) via the RStudio^®^ interface (2024.12.1), with the summarytools statistical package.

### Temporal Trends in Tuberculosis Cases

The Seasonal-Trend Decomposition using Loess (STL) was applied, a robust method that decomposes time series into three components using locally weighted regression: trend, seasonality, and residual^14^. The study focused exclusively on the analysis of the trend component, allowing the temporal behavior of tuberculosis morbidity and mortality variables to be characterized.

To assess the recrudescence, two milestones were defined for the pandemic period, based on epidemiological evidence:

- Milestone A (August/2021): a period in which 50% of the Brazilian population received at least one dose of the COVID-19 vaccine, 22% completed the vaccination schedule, and a 40% reduction in COVID-19 mortality was observed^15^;
- Milestone B (April/2022): corresponds to the decree closing the Public Health Emergency of National Concern (ESPIN) in Brazil^11^.

### Interrupted Time Series

The interrupted time series (ITS) technique was used, conducted through autoregressive integrated moving average (ARIMA) models, according to the Box-Jenkins method^16^, in order to assess the impact of the COVID-19 pandemic on the selected outcomes. The approach adopted followed that of the model proposed by Schaffer et al.^17^(2021), which recommends the use of ARIMA models for evaluating large-scale interventions in public health, based on time series.

Initially, the stationarity of the series was assessed through visual inspection, statistical tests and analysis of the autocorrelation (ACF) and partial autocorrelation (PACF) functions. When necessary, seasonal and non-seasonal differencing was applied to stabilize the average and remove trends or seasonal patterns.

The order of the autoregressive (p), differencing (d), and moving average (q) components, as well as the seasonal components (P, D, Q), was based on the inspection of the ACF and PACF plots and the minimization of information criteria (Akaike Information Criterion - AIC)^16^. Model fitting was performed iteratively until the residuals exhibited white noise behavior, verified using the Ljung-Box, Box-Pierce, Turning Point, T-test, and Kolmogorov-Smirnov tests.

To estimate the impact of the pandemic, intervention indicator variables representing abrupt changes (step) and changes in the slope (or ramp) of the trend were included, with March 2020 defined as the starting point of the intervention. The statistical significance of the estimated coefficients was assessed using 95% confidence intervals (95%CI).

### Spatial Association of Tuberculosis

An exploratory approach based on the Moran’s I and Getis-Ord Gi* tests was used to assess spatial autocorrelation. The global Moran’s index (Moran’s I) was applied to verify the presence, strength and direction of spatial autocorrelation between the state’s municipalities, based on a Queen-type neighborhood matrix of order 1. This index ranges from -1 to +1, with positive values indicating direct spatial association (grouping of similar values), while negative values indicate inverse association and values close to zero suggest spatial randomness. Statistical significance was considered for p < 0.05^18^. The Moran’s I analysis was carried out using the GeoDa^®^ software, version 1.22.0.14^19^.

On a local scale, the Getis-Ord Gi* test was used to detect significant spatial clusters and identify hotspots and coldspots. The Gi*(d) value is expressed by a Z score, which can vary from -3 to +3, indicating significant clusters of low and high values, respectively. The higher the value of the Z score, the stronger the grouping of high rates (hotspots), while negative values indicate groupings of low rates *(*coldspots). Values close to zero indicate no significant clustering^20^. In the Gi* analyses, three levels of statistical significance were considered: 90%, 95%, and 99%, which allowed for a more refined classification of the intensity of spatial clusters.

The territorial grid of the state’s municipalities was obtained from the IBGE collection^12^, and the choropleth maps were drawn up using ArcGIS^®^ software version 10.5.

### Ethical Aspects

The study was approved by the Research Ethics Committee of the Escola de Enfermagem de Ribeirão Preto, Universidade de São Paulo (EERP/USP), under the Certificate of Submission for Ethical Appraisal (CAAE) number 77580424.5.0000.5393, in accordance with Resolutions No. 466/2012 and No. 510/2016 of the National Health Council.

## RESULTS

### Descriptive Analysis

Table 1 describes the sociodemographic and clinical-epidemiological variables of notified tuberculosis cases. Between 2015 and 2023, 196,950 cases of tuberculosis were reported in the state of São Paulo. Of this total, 55.9% (n = 110,051) occurred in the pre-pandemic period, 23.3% (n = 45,926) during the pandemic, and 20.8% (n = 40,973) in the post-pandemic period, showing a drop in recent notifications. The profile of cases remained predominantly male (n = 142,767; 72.5%), with a mean age of 39 years.

**Table 1:** Sociodemographic and clinical characteristics of people diagnosed with tuberculosis in the state of São Paulo from 2015–2023.

Characteristic	Cumulative (2015–2023)	Pre-pandemic (01/2015–01/2020)	Pandemic (02/2020–04/2022)	Post-pandemic (05/2022–12/2023)
	n (%)	n (%)	n (%)	n (%)
Total	196,950 (100.0)	110,051 (55.9)	45,926 (23.3)	40,973 (20.8)
Sex				
Male	142,767 (72.5)	80,358 (73.0)	32,975 (71.8)	29,434 (71.8)
Female	54,183 (27.5)	29,693 (27.0)	12,951 (28.2)	11,539 (28.2)
Age group (years)				
0–14	4,500 (2.3)	2,563 (2.3)	916 (2.0)	1,021 (2.5)
15–29	58,136 (29.5)	33,936 (30.8)	13,164 (28.7)	11,036 (27.0)
30–39	47,206 (24.0)	26,479 (24.1)	11,001 (24.0)	9,726 (23.7)
40–49	35,648 (18.1)	19,130 (17.4)	8,561 (18.6)	7,957 (19.4)
50–59	26,311 (13.4)	14,579 (13.2)	6,191 (13.5)	5,541(13.5)
≥ 60	24,852 (12.6)	13,184 (12.0)	6,032 (13.1)	5,636 (13.8)
No information	297 (0.1)	180 (0.2)	61 (0.1)	56 (0.1)
Race/skin color				
White	71,171 (36.1)	44,017 (40.0)	16,540 (36.0)	10,614 (25.9)
Black	20,364 (10.3)	11,773 (10.7)	5,172 (11.3)	3,419 (8.4)
Yellow	1,344 (0.7)	787 (0.72)	345 (0.8)	212 (0.5)
Brown	67,897 (34.5)	39,298 (35.7)	17,124 (37.3)	11,475 (28.0)
Indigenous	404 (0.2)	282 (0.3)	69 (0.2)	53 (0.1)
No information	35,770 (18.2)	13,894 (12.6)	6,676 (14.5)	15,200 (37.1)
Level of education (years)				
No schooling	4,653 (2.4)	2,813 (2.6)	902 (2.0)	938 (2.3)
1–3	13,137 (6.7)	7,888 (7.2)	2,902 (6.3)	2,347 (5.7)
4–7	50,857 (25.8)	30,143 (27.4)	11,155 (24.3)	9,559 (23.3)
8–11	57,866 (29.4)	32,473 (29.5)	13,595 (29.6)	11,798 (28.8)
12–14	13,030 (6.6)	6,845 (6.2)	3,261 (7.1)	2,924 (7.1)
≥ 15	5,297 (2.7)	2,975 (2.7)	1,268 (2.8)	1,054 (2.6)
No information	52,110 (26.5)	26,914 (24.5)	12,843 (28)	12,353 (30.1)
Type of entry				
New case	159,683 (81.0)	90,463 (82.2)	36,895 (80.3)	32,325 (78.9)
Recurrence	19,251 (9.8)	10,847 (9.9)	4,512 (9.8)	3,892 (9.5)
Retreatment after loss to follow-up	15,376 (7.9)	7,575 (6.9)	3,708 (8.1)	4,093 (10)
Retreatment after failure/resistance	1,445 (0.7)	796 (0.7)	353 (0.8)	296 (0.7)
Retreatment after change of regimen	1,195 (0.6)	370 (0.3)	458 (1.0)	367 (0.9)
Histopathology				
AFB positive	3,638 (1.9)	1,990 (1.8)	930 (2.0)	718 (1.8)
Suggestive of tuberculosis	8,305 (4.2)	5,017 (4.6)	1,907 (4.2)	1,381 (3.4)
Not performed	141,752 (72.0)	78,827 (71.6)	32,343 (70.4)	30,582 (74.6)
No information	43,255 (22.0)	24,217 (22.0)	10,746 (23.4)	8,292 (20.2)
Smear microscopy				
Positive	92,581 (47.0)	54,098 (49.2)	20,779 (45.2)	17,704 (43.2)
Negative	40,337 (20.5)	23,990 (21.8)	8,615 (18.8)	7,732 (18.9)
In progress	760 (0.4)	296 (0.3)	135 (0.3)	329 (0.8)
Not performed	61,423 (31.2)	30,819 (28)	15,903 (34.6)	14,701 (35.9)
No information	1,849 (0.9)	848 (0.8)	494 (1.1)	507 (1.2)
Culture				
Positive	67,676 (34.4)	39,527 (35.9)	15,015 (32.7)	13,134 (32.1)
Negative	23,873 (12.1)	13,773 (12.5)	5,598 (12.2)	4,502 (11.0)
In progress	2,464 (1.3)	949 (0.9)	342 (0.7)	1,173 (2.9)
Not performed	97,287 (49.4)	52,521 (47.7)	23,758 (51.7)	21,008 (51.3)
No information	5,650 (2.9)	3,281 (3.0)	1,213 (2.6)	1,156 (2.8)
Human Immunodeficiency Virus (HIV) test				
Positive	18,018 (9.2)	10,405 (9.5)	3,875 (8.4)	3,738 (9.1)
Negative	160,738 (81.6)	89,410 (81.2)	38,049 (82.9)	33,279 (81.2)
Not performed	12,550 (6.4)	7,443 (6.8)	2,727 (5.9)	2,380 (5.8)
In progress	983 (0.5)	432 (0.4)	179 (0.4)	372 (0.9)
No information	4,661 (2.4)	2,361 (2.2)	1,096 (2.4)	1,204 (2.9)
Susceptibility test				
Yes	48,681 (24.7)	24,603 (22.4)	12,693 (27.6)	11,385 (27.8)
No	113,269 (57.5)	56,452 (51.3)	29,856 (65.0)	26,961 (65.8)
No information	35,000 (17.8)	28,996 (26.4)	3,377 (7.4)	2,627 (6.4)
X-ray examination				
Normal	10,199 (5.2)	5,994 (5.5)	2,283 (5.0)	1,922 (4.7)
Suspected tuberculosis	95,607 (48.5)	55,332 (50.3)	21,705 (47.3)	18,570 (45.3)
Suspected tuberculosis with cavity	25,633 (13.0)	14,539 (13.2)	5,977 (13.0)	5,117 (12.5)
Other pathology	2,280 (1.2)	1,335 (1.2)	446 (1.0)	499 (1.2)
Not performed	47,213 (24.0)	24,259 (22.0)	11,628 (25.3)	11,326 (27.6)
No information	16,018 (8.1)	8,592 (7.8)	3,887 (8.5)	3,539 (8.6)
Clinical form				
Pulmonary	163,973 (83.3)	91,498 (83.1)	38,126 (83.0)	34,349 (83.8)
Extrapulmonary	26,388 (13.4)	14,989 (13.6)	6,196 (13.5)	5,203 (12.7)
Pulmonary + extrapulmonary	5,991 (3.0)	3,256 (3.0)	1,449 (3.2)	1,286 (3.1)
Widespread	588 (0.3)	298 (0.3)	155 (0.3)	135 (0.3)
No information	10 (0)	10 (0)	0 (0)	0 (0)
Outcome				
Loss to follow-up	29,634 (15.1)	14,228 (12.9)	7,763 (16.9)	7,643 (18.7)
Cure	142,387 (72.3)	84,720 (77.0)	32,486 (70.7)	25,181 (61.5)
Failure/resistance	1,475 (0.8)	804 (0.7)	358 (0.8)	313 (0.8)
Change of scheme	1,426 (0.7)	465 (0.4)	532 (1.2)	429 (1.1)
Death	15,760 (8.0)	8,292 (7.5)	3,952 (8.6)	3,516 (8.6)
Transfer	1,718 (0.9)	882 (0.8)	444 (1.0)	392 (1.0)
No information	4.550 (2.3)	660 (0.6)	391 (0.9)	3,499 (8.5)
Rapid molecular test for tuberculosis (TRM-TB)				
Detectable sensitive to rifampicin	68,948 (35.0)	32,635 (29.7)	18,270 (39.8)	18,043 (44.0)
Detectable resistant to rifampicin	2,043 (1.0)	1,038 (0.9)	509 (1.1)	496 (1.2)
Detectable rifampicin undetermined	1,367 (0.7)	247 (0.2)	502 (1.1)	618 (1.5)
Not detectable	13,162 (6.7)	6,654 (6.1)	3,145 (6.9)	3,363 (8.2)
Inconclusive	513 (0.3)	269 (0.2)	118 (0.3)	126 (0.3)
Not performed	80,146 (40.7)	48,520 (44.1)	17,580 (38.3)	14,046 (34.3)
No information	30,771 (15.6)	20,688 (18.8)	5,802 (12.6)	4,281 (10.5)
Antiretroviral therapy				
Yes	8,579 (4.4)	4.164 (3.8)	2,166 (4.7)	2,249 (5.5)
No	2,236 (1.1)	1,149 (1.0)	571 (1.2)	516 (1.3)
No information	186,135 (94.5)	104,738 (95.2)	43,189 (94.0)	38,208 (93.3)
Homeless population				
Yes	10,019 (5.1)	5,169 (4.7)	2,417 (5.3)	2,433 (5.9)
No	186,931 (94.9)	104,882 (95.3)	43,509 (94.7)	38,540 (94.1)
Incarcerated population				
Yes	23,904 (12.1)	15,800 (14.4)	4,734 (10.3)	3,370 (8.2)
No	173,046 (87.9)	94,251 (85.6)	41,192 (89.7)	37,603 (91.8)
Immigrants				
Yes	1,395 (0.7)	872 (0.8)	337 (0.7)	186 (0.5)
No	119,649 (60.8)	76,401 (69.4)	27,635 (60.2)	15,613 (38.1)
No information	75,906 (38.5)	32,778 (29.8)	17,954 (39.1)	25,174 (61.4)
Aids				
Yes	16,749 (8.5)	9,714 (8.8)	3,599 (7.8)	3,436 (8.4)
No	180,201 (91.5)	100,337 (91.2)	42,327 (92.2)	37,537 (91.6)
Diabetes				
Yes	13,421 (6.8)	6,953 (6.3)	3,575 (7.8)	2,893 (7.1)
No	183,529 (93.2)	103,098 (93.7)	42,351 (92.2)	38,080 (92.9)
Alcoholism				
Yes	39,259 (19.9)	20,861 (19.0)	9,354 (20.4)	9,044 (22.1)
No	157,691 (80.1)	89,190 (81.0)	36,572 (79.4)	31,929 (77.9)
Smoking				
Yes	51,802 (26.3)	27,335 (24.8)	12,478 (27.2)	11,989 (29.3)
No	145,148 (73.7)	82,716 (75.2)	33,448 (72.8)	28,984 (70.7)
Mental illness				
Yes	3,024 (1.5)	1,639 (1.5)	727 (1.6)	658 (1.6)
No	193,926 (98.5)	108,412 (98.5)	45,199 (98.4)	40,315 (98.4)
Drug addiction				
Yes	39,052 (19.8)	20,745 (18.9)	9,118 (19.9)	9,189 (22.4)
No	157,898 (80.2)	89,306 (81.2)	36,808 (80.1)	31,784 (77.6)
Other immune disease				
Yes	2,359 (1.2)	1,316 (1.2)	556 (1.2)	487 (1.2)
No	194,591 (98.8)	108,735 (45.4)	45,370 (98.8)	40,486 (98.8)

AFB: Acid-fast bacillus.

There was a significant change in the racial profile: while the White category was predominant before the pandemic (n = 44,017; 40.0%), there was a reversal in the pandemic (n = 17,124; 37.3%), and post-pandemic (n = 11,475; 28%) periods, with a relative increase in cases among brown people. Also noteworthy is the sharp increase in missing data for race/skin color and schooling in the post-pandemic, which may indicate weaknesses in surveillance and hinder more detailed analyses of inequalities.

Most cases were classified as new occurrences, but the proportion fell from 82.2% (n = 90,463) in the pre-pandemic to 78.9% (n = 32,325) in the post-pandemic period, whereas cases of re-treatment after loss to follow-up increased from 6.9% (n = 7,575) to 10.0% (n = 4,093), suggesting that the pandemic had an impact on continuity of care. The pulmonary form remained predominant (n = 163,973; 83.3%).

The outcomes reveal a worsening scenario: the cure rate fell from 77.0% (n = 84,720) in the pre-pandemic to 61.5% (n = 25,181) in the post-pandemic period, whereas loss to follow-up jumped from 12.9% (n = 14,228) to 18.7% (n = 7,643). This indicates a breakdown in adherence and a greater risk of transmission and resistance.

Among comorbidities, the prevalence of people living with HIV remained stable throughout the period, but there was an increase in cases associated with diabetes during the pandemic. Social risk factors, such as alcoholism, smoking, and drug use, showed consistent growth in the post-pandemic period, indicating a worsening of social vulnerabilities.

In terms of vulnerable populations, the significant reduction in the notification of cases among incarcerated people (from 14.4% [n = 15,800] to 8.2% [n = 3,370]) is noteworthy, whereas cases among homeless people increased slightly from 4.7% (n = 5,169) to 5.9% (n = 2,433), suggesting possible shortcomings in screening and access to diagnosis in these populations.

### Time Trends and Interrupted Time Series

**Figure 1:**
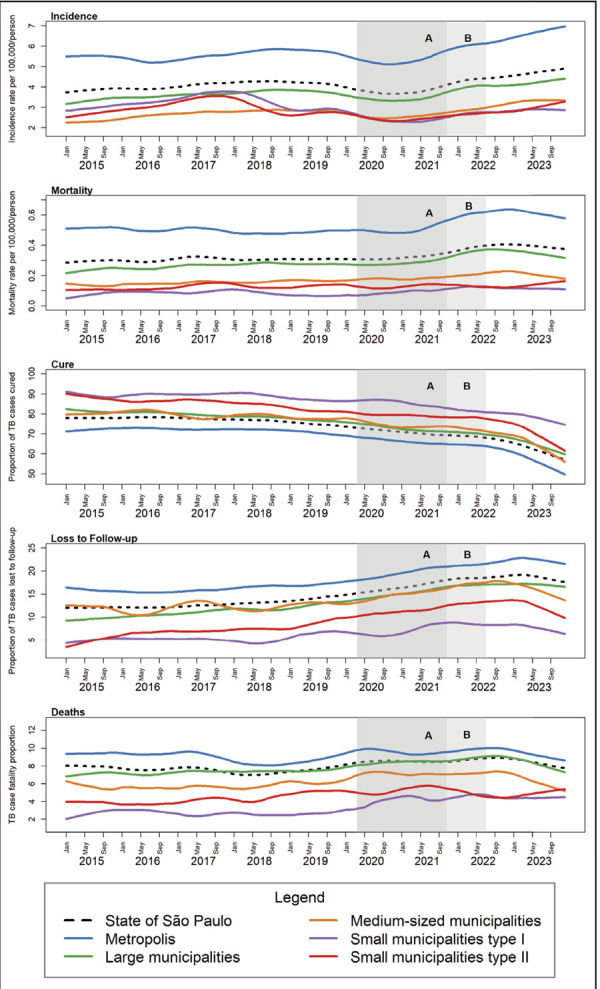
Trends in incidence and mortality rates and tuberculosis treatment outcomes in municipalities in the state of São Paulo, according to population size categories, 2015 to 2023.

Figure 1 shows the time trends in tuberculosis incidence, mortality, and treatment outcomes.

An analysis of the incidence and mortality trends shows important changes from 2020 onwards. Between the beginning of the pandemic and 2021, the notification of cases shows a reduction among all sizes of municipality and especially in metropolitan areas. From 07/2021 onwards, the notification of cases starts to increase gradually, reaching historic highs at the end of 2023 in the state and among large municipalities and metropolises. Smaller municipalities (medium and small I and II) showed similar trends, but with more discreet variations over time.

Similarly, there was an increase in mortality rates after 2021, reaching the highest monthly values in the series in 2022 and starting to fall at the end of 2023.

Among the treatment outcomes, there has been a marked reduction in the proportion of cases closed as cured, from the beginning of 2020 to the end of 2023. Conversely, there was an increase in the proportion of cases lost to follow-up, which had already shown signs of increasing before the start of the pandemic.

Table 2 shows the results of the STI analysis, with the respective adjusted ARIMA models and estimates of the intervention effects (step and slope). In addition, the results of the Ljung- Box test, used to check for the presence of autocorrelation in the residuals, and the Mean Absolute Percentage Error (MAPE), which assesses the accuracy of the models based on the average of the absolute percentage errors between the predicted values and the observed values, are presented.

**Table 2:** ARIMA interrupted time series modeling to evaluate trends in tuberculosis incidence, mortality, and treatment outcomes in the state of São Paulo.

Indicator/municipal size	ARIMA model	Step (95%CI)	Slope (95%CI)	MAPE	Ljung-Box (p-value)
Incidence					
State of São Paulo	(0,0,2)(1,0,0)	-0.69 (-0.94 to -0.44)	0.034 (0.025 to 0.043)	5.62	27.44 (0.28)
Large	(0,0,0)(1,0,0)	-0.54 (-0.76 to -0.33)	0.030 (0.022 to 0.037)	6.69	24.51 (0.43)
Medium-sized	(0,0,0)(1,0,0)	-0.40 (-0.67 to -0.12)	0.025 (0.016 to 0.035)	12.23	17.02 (0.85)
Metropolis	(0,0,5)	-0.91 (-1.31 to -0.51)	0.052 (0.038 to 0.065)	6.83	20.11 (0.69)
Small I	(2,0,0)(1,0,1)	-0.70 (-1.36 to -0.05)	0.006 (-0.016 to 0.029)	13.58	33.57 (0.09)
Small II	(0,1,1)(2,0,0)	-0.22 (-0.69 to 0.25)	0.023 (-0.025 to 0.070)	10.59	27.45 (0.28)
Mortality					
State of São Paulo	(2,0,2)	-0.0098 (-0.0371 to 0.0174)	0.0026 (0.0016 to 0.0035)	8.44	15.10 (0.92)
Large	(1,0,1)	0.0023 (-0.0415 to 0.0461)	0.0021 (0.0007 to 0.0035)	12.51	16.52 (0.87)
Medium-sized	(2,1,3)	0.0294 (-0.0088 to 0.0676)	0.0005 (-0.0008 to 0.0018)	32.91	13.65 (0.95)
Metropolis	(0,1,1)	-0.0206 (-0.0688 to 0.0276)	0.0039 (0.0022 to 0.0056)	11.44	28.13 (0.25)
Small I	(2,1,0)	-0.0409 (-0.1653 to 0.0836)	0.0000 (-0.0119 to 0.0119)	∞	32.47 (0.11)
Small II	(0,1,1)	0.5725 (-1.8004 to 2.9455)	-0.0016 (-0.0504 to 0.0472)	50.37	19.86 (0.70)
Cure					
State of São Paulo	(1,1,0)	1.946 (-1.498 to 5.389)	-0.672 (-1.099 to -0.246)	1.95	19.74 (0.71)
Large	(1,0,4)	1.961 (-2.828 to 6.749)	-1.036 (-1.617 to -0.455)	2.67	12.80 (0.97)
Medium-sized	(0,0,3)	0.040 (-5.712 to 5.792)	-0.444 (-0.642 to -0.245)	5.32	13.16 (0.96)
Metropolis	(2,0,0)	2.480 (-1.815 to 6.774)	-0.573 (-0.779 to -0.368)	2.80	13.04 (0.97)
Small I	(1,1,1)(2,0,0)	1.726 (-1.424 to 4.875)	-0.294 (-0.401 to -0.187)	3.67	18.41 (0.78)
Small II	(1,0,1)	0.030 (-5.376 to 5.436)	-0.416 (-0.625 to -0.206)	4.46	16.24 (0.88)
Loss to follow-up					
State of São Paulo	(1,1,1)	0.507 (-1.134 to 2.148)	0.063 (-0.024 to 0.149)	5.91	14.24 (0.94)
Large	(1,1,2)	1.261 (-0.451 to 2.973)	0.042 (-0.050 to 0.134)	8.16	15.99 (0.89)
Medium-sized	(0,1,1)	2.784 (0.817 to 4.750)	0.040 (-0.027 to 0.107)	18.66	24.09 (0.46)
Metropolis	(0,0,1)	2.057 (0.756 to 3.358)	0.106 (0.061 to 0.150)	7.31	15.32 (0.91)
Small I	(0,1,3)(0,0,1)	-0.940 (-4.564 to 2.684)	0.032 (-0.109 to 0.173)	∞	31.55 (0.14)
Small II	(0,1,1)	0.876 (-2.377 to 4.130)	0.081 (-0.026 to 0.189)	30.15	16.61 (0.86)
Deaths					
State of São Paulo	(0,1,1)	1.339 (0.852 to 1.826)	-0.010 (-0.027 to 0.006)	7.55	22.48 (0.55)
Large	(1,1,2)	1.441 (0.838 to 2.044)	-0.007 (-0.028 to 0.014)	9.85	20.33 (0.68)
Medium-sized	(1,0,1)	1.979 (1.552 to 2.406)	-0.030 (-0.047 to -0.013)	31.99	25.72 (0.37)
Metropolis	(2,1,2)	1.695 (0.474 to 2.916)	-0.048 (-0.130 to 0.035)	10.12	17.32 (0.83)
Small I	(0,1,1)	0.613 (-0.748 to 1.973)	0.026 (-0.020 to 0.073)	∞	19.10 (0.75)
Small II	(0,1,1)	0.573 (-1.800 to 2.945)	-0.002 (-0.050 to 0.047)	50.37	22.95 (0.52)

95%CI: 95% confidence interval; MAPE: mean absolute percentage error; ARIMA: autoregressive integrated moving average models; ∞: infinity.

The application of the technique revealed that the COVID-19 pandemic had statistically significant impacts on different tuberculosis indicators in the state of São Paulo, varying according to municipal population size.

For the incidence of tuberculosis, there was an immediate reduction among all groups of municipalities, with the exception of small municipalities. The metropolises showed the greatest reduction in the step at 0.91 cases per 100,000 inhabitants (95%CI: -1.31 to -0.51), possibly reflecting underreporting of cases in the first months of the pandemic. Larger municipalities (metropolis, large, and medium-sized) showed upward trends after the pandemic, reaching 0.052 cases per 100,000 inhabitants/month in the metropolises.

With regard to tuberculosis mortality, there was an increasing trend over time in the state after the start of the pandemic (slope: 0.0026; 95%CI: 0.0016 to 0.0035), indicating a progressive worsening of the indicator. This same pattern was identified in large municipalities and metropolises.

For the proportion of cures, no immediate changes were detected in the series with the start of the pandemic. On the other hand, all size categories showed downward trends, with large municipalities standing out with a reduction of 1.03% per month (95%CI: -1.62 to -0.46).

In the loss to follow-up indicator, there was an immediate increase in medium-sized municipalities (step: 2.783; 95%CI: 0.8171 to 4.750) and in metropolitan regions (step: 2.056; 95%CI: 0.7559 to 3.357). The models also showed an increasing trend over time in metropolitan areas (slope: 0.1058; 95%CI: 0.0614 to 0.1502).

Deaths from tuberculosis were also significantly affected: there was an immediate increase in the state (step: 1.339; 95%CI: 0.852 to 1.826), in large municipalities (step: 1.441; 95%CI: 0.838 to 2.044), medium-sized municipalities (step: 1.979; 95%CI: 1.552 to 2.406), and in metropolitan regions (step: 1.695; 95%CI: 0.474 to 2.916).

The models adjusted for all the series were those with the best predictive capacity and the lowest AIC value compared to the other options tested. In addition, all the models selected had non-autocorrelated residuals with a normal distribution, meeting the fundamental modeling assumptions. Thus, the adjusted models proved to be valid for predictive and analytical purposes.

### Spatial Association of Tuberculosis Incidence and Mortality

**Figure 2:**
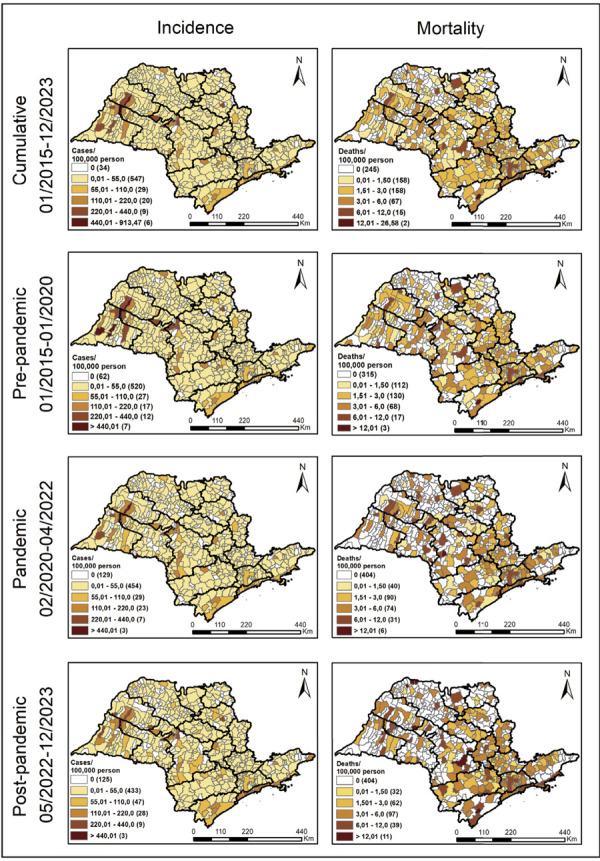
Spatial distribution of tuberculosis incidence and mortality rates. São Paulo, Brazil, 2015 to 2023.

The spatial pattern of tuberculosis incidence in the state of São Paulo is heterogeneous and has remained consistent across the study periods, with clusters of higher rates in the regions of Presidente Prudente, Araçatuba, Bauru, and Baixada Santista. Notably, when comparing the pandemic period with the post-pandemic period, there was an increase in the number of municipalities with an incidence of 110 cases per 100,000 inhabitants (Figure 2). Tuberculosis mortality rates showed a widely distributed increase throughout the state after the pandemic. At the same time, the number of municipalities not reporting deaths increased (Figure 2).

Over the period analyzed, positive spatial autocorrelation was observed, with variations in intensity according to the phase of the pandemic. For the incidence rate, the global autocorrelation measured by the global Moran’s index showed a moderate value in the cumulative period (2015–2023), with Moran’s I = 0.15 (p < 0.01). In the pre-pandemic period, the value was slightly higher, Moran’s I = 0.19 (p < 0.01), indicating a more pronounced pattern of spatial clustering.

During the pandemic period, autocorrelation decreased considerably, with Moran’s I = 0.05 (p = 0.01), suggesting a weakening of the spatial structure. In the post-pandemic period, there was a slight increase, with Moran’s I = 0.09 (p < 0.01).

With regard to mortality, positive spatial autocorrelation was also identified over all periods. The cumulative value was 0.13 (p < 0.01). In the pre-pandemic period, autocorrelation was similar, with Moran’s I = 0.12 (p < 0.01). During the pandemic, the intensity of spatial clustering was lower, with Moran’s I = 0.051 (p = 0.02), and increased slightly again in the post-pandemic period, reaching 0.08 (p < 0.01).

**Figure 3:**
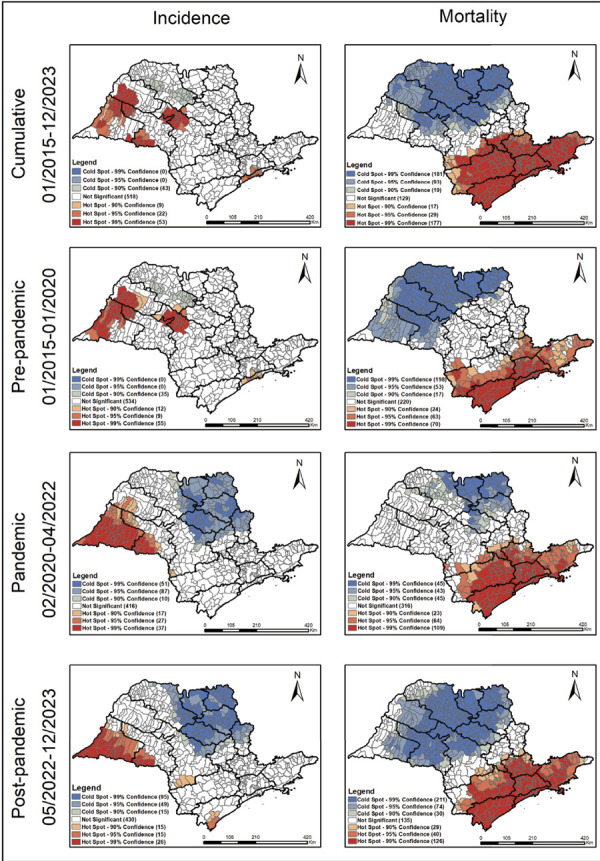
Municipalities in the state of São Paulo with local Getis-Ord Gi* spatial association for tuberculosis incidence and mortality rates. Brazil, 2015 to 2023.

The results of the Gi* analysis (Figure 3) show the formation of hotspots of high incidence of tuberculosis in the state of São Paulo, showing the persistence of a high burden of the disease in the Presidente Prudente region, dispersing to the other municipalities with high rates over time.

In the Marília and Bauru region, there was a change in the spatial pattern: while the hotspots were concentrated in the northernmost municipalities in the pre-pandemic period, the concentration of cases began to occur after the pandemic in the southern municipalities of the region, suggesting a geographical dispersion of transmission. Also in the post-pandemic period, a new hotspot emerged in the region of Registro, indicating a possible local recrudescence in the transmission of the disease.

With regard to tuberculosis mortality hotspots, the regions of Taubaté, Registro, Baixada Santista, and Grande São Paulo showed a high mortality rate in all the periods analyzed. The Sorocaba and Campinas regions showed clusters in the pre-pandemic period, which dispersed throughout the municipalities and became more evident after the pandemic, suggesting a worsening of the epidemiological scenario (Figure 3).

## DISCUSSION

The study showed that the post-pandemic recrudescence of tuberculosis in São Paulo is characterized by an increase in incidence, loss to follow-up, an increase in mortality and a reduction in cure rates. Clusters were identified in areas with low primary health care coverage, indicating that local contextual factors and the fragmentation of health services played an important role in the dynamics of the disease.

The findings are in line with evidence from other national and international contexts. While studies in cities such as Rio de Janeiro^21^and in countries with a high tuberculosis burden^2,22^have reported a sharp drop in notifications and difficulties in recovering post-pandemic indicators, these findings show that, in São Paulo, underreporting and worsening of outcomes persist during this period, distributed unevenly across the state.

In countries such as Australia and Singapore, the impacts were less intense as compared to other countries in Asia and the Americas. A multinational study pointed out that the implementation of surveillance strategies and the use of digital health technologies may have contributed to mitigating the negative effects of the pandemic in these contexts^23^.

The recrudescence of tuberculosis was also observed in other countries, and in France there was a reduction in the detection of cases in early 2020, followed by an increase in mortality in the same period, a behavior different from that observed in the study, in which the increase in mortality occurred from 2021^24^.

The application of the STL decomposition method identified changes in the trends of incidence and mortality rates, as well as in the operational indicators of cure, death, and loss to follow-up, over the different periods analyzed.

The choice of time frames based on COVID-19 vaccination coverage and the end of the national health emergency allowed for a more precise analysis of the direct and indirect effects of the pandemic on the dynamics of tuberculosis, in line with international recommendations for time series studies in public health^25^.

With regard to incidence, although it was reduced at the start of the pandemic, the study reveals strong evidence of underreporting, especially during the pandemic, which compromised the ability of services to identify and properly notify cases, as has been shown in other contexts^26^.

The lack of timely diagnosis and treatment may partly explain the upward trend in cases, and therefore recrudescence, observed from the second half of 2021, reaching historically high levels by the end of 2023, especially in large urban centers and metropolitan regions.

The pattern suggests an accumulation of undiagnosed people during the pandemic period and a subsequent recrudescence in transmission. This may be one of the factors linked to the increase in tuberculosis mortality in the state of São Paulo from the end of 2020, since access to and follow-up of treatment also showed sharp drops and downward trends after the pandemic.

The findings point to underreporting and worsening outcomes, associated with a consistent increase in social risk factors, such as alcoholism, smoking, and drug use, in the post-pandemic period^7^, thus reinforcing the need for integrated policies that consider the social determinants of health.

In the analysis of the recrudescence of tuberculosis, there was a significant increase in drug (22.4%), alcohol (22.1%), and tobacco (29.3%) use in the post-pandemic period, possibly related to stress and changes in living conditions during the COVID-19 pandemic.

This increase may be associated with cases lost to follow-up and disease recurrence^27,28^. This situation reinforces the need for intersectoral actions and systemic and integrated approaches, from health, social assistance, and other sectors, with the possibility of including and developing harm reduction programs and support for cessation of substance use^28,29^.

Another important result was the reduction in case detection (from 14.4% in the pre-pandemic to 8.2% in the post-pandemic period) among incarcerated people in the post-pandemic, which is possibly related to underreporting. It is possible that this drop is linked to the lack of policies and prioritization of populations^8,29^.

Spatial analysis showed the formation and intensification of clusters when considering incidence. This may be associated, in addition to detection issues, with worsening living conditions for the population, such as high population density, unhealthy conditions, and weaknesses in the quality of the services provided in these locations, especially primary health care^30,31^.

In the Presidente Prudente region, clusters of high incidence were observed, with the presence of large prison complexes^32^and low population density^33^, suggesting that health surveillance and care in these environments are still insufficient to contain transmission.

With regard to tuberculosis mortality, clusters were observed, mainly in regions with high population density and low primary health care coverage, such as the Baixada Santista, Grande São Paulo, and Taubaté regions^33^. These areas concentrate important economic centers with intense movement of people, but also have high levels of social inequality.

In 2021, the São Paulo Metropolitan Region had more than 1 million families living in poverty —corresponding to 54% of all families in this situation in the state^34^. The discrepancy observed between the expected demographic profile and the casuistic data, with a predominance of cases among people of White race/skin color, may be related to the state’s population distribution and underreporting in historically more vulnerable groups, aggravated by the absence of complete data on race/skin color and schooling in the post pandemic period. The high proportion of missing data limits more detailed analyses of equity and demands improvements to information systems^37^.

The study’s limitations include the use of secondary data, which can hide intraregional heterogeneities and mask potential silent areas, since the study uses data recorded by health services and reflects only known cases.

In addition, the absence of complete information on sociodemographic and clinical variables in some of the records compromises the accuracy of the analysis, especially with regard to the profile of cases and treatment outcomes. We recommend exercising caution when generalizing the results and that qualitative studies be carried out to gain a deeper understanding of the local determinants of the clusters identified in the study.

## CONCLUSION

The analysis of the recrudescence of tuberculosis in the state of São Paulo after the COVID-19 pandemic showed a significant worsening of the epidemiological scenario. There was a reduction of 1.03% per month in cure rates in large municipalities and a significant increase in loss to follow-up, which rose to 2.78% per month in medium-sized municipalities. At the same time, tuberculosis mortality showed an upward trend in the post-pandemic period, reflecting unequal impacts in the different territories.

The findings show persistent clusters and the emergence of new tuberculosis epicenters in the state, reflecting a global scenario documented by the World Health Organization. This reinforces the urgency of realigning with the “End TB” strategy, whose essential actions include investment in research and innovation, preventive treatment, early diagnosis (preferably molecular-based), timely treatment, monitoring and adherence strategies, and social protection systems. There is also the need to prepare for future emergencies, with resilient surveillance systems capable of maintaining essential tuberculosis services during crises.

## Data Availability

All data supporting the findings of the study are contained in the manuscript. Additional detailed information and raw data will be made available upon request to the corresponding author.
