# Supplementary material for: Recrudescence of tuberculosis in the state of São Paulo post-COVID-19: trends and clusters
Source: Rev Saude Publica. 2026 Jan 9;59:e50. doi: 10.11606/s1518-8787.2025059006987 (PMC12713529; doi:10.11606/s1518-8787.2025059006987)
Supplement: Translation

# Translation: Recrudescimento da tuberculose no estado de São Paulo pós-covid-19: tendências e clusters

**Authors:** Reginaldo Bazon Vaz Tavares, Nathalia Zini, Yan Mathias Alves, André Luiz Teixeira Vinci, Natacha Martins Ribeiro, Ariela Fehr Tártaro, Valter Chicalo António Caripa, Maria Eduarda Pagano Pelodan, Clara Ferreira de Souza, Aline Aparecida Monroe, Jaqueline Garcia de Almeida Ballestero, Ione Carvalho Pinto, Pedro Fredemir Palha, Ricardo Alexandre Arcêncio

**Affiliations:** I Universidade de São Paulo. Escola de Enfermagem de Ribeirão Preto. Departamento de Enfermagem Materno-Infantil e Saúde Pública. Ribeirão Preto, SP, Brasil

**Keywords:** Tuberculose, COVID-19, Recrudescimento, Tendências, Saúde Pública

## Abstract

**OBJETIVO:**

Analisar o recrudescimento da tuberculose no estado de São Paulo após a pandemia de covid-19, identificando tendências temporais e *clusters* espaciais da doença.

**MÉTODOS:**

Estudo ecológico de casos de tuberculose notificados no TBWeb em todos os municípios paulistas entre janeiro de 2015 e dezembro de 2023. Foram aplicadas técnicas de decomposição temporal, análise de séries temporais interrompidas por mês e análise espacial por municípios (índice global de Moran e Getis-Ord Gi*) para identificar tendências, mudanças abruptas associadas à pandemia e *clusters* de alta incidência, mortalidade e desfechos do tratamento. Os períodos pré-pandêmico (01/2015 a 01/2020), pandêmico (02/2020 a 04/2022) e pós-pandêmico (05/2022 a 12/2023) foram analisados separadamente.

**RESULTADOS:**

Observou-se o recrudescimento da tuberculose no período pós-pandêmico, com aumento de 21,2% no número de municípios com incidência > 110 casos por 100 mil habitantes. Verificou-se aumento progressivo da tendência de mortalidade em 0,0026 (IC95% 0,0016 a 0,0035) mortes por 100.000 habitantes ao mês após a pandemia. Enquanto houve queda gradual de 0,67% (IC95% -1,099 a -0,246) ao mês na proporção de cura após a pandemia. Foi constatada a persistência de *clusters* de alta incidência na região de Presidente Prudente em todos os períodos, e novos *clusters* em Marília e Registro após a pandemia. As áreas com altos índices de mortalidade persistiram nas regiões de Taubaté, Baixada Santista, Grande São Paulo, Registro, Sorocaba e Campinas em todos os períodos.

**CONCLUSÃO:**

O recrudescimento da tuberculose em São Paulo no contexto pós-pandemia evidencia a necessidade de estratégias direcionadas para diagnóstico precoce, fortalecimento do tratamento e monitoramento intensivo em regiões identificadas como *clusters*, especialmente aquelas com populações vulneráveis e desafios estruturais dos serviços de saúde.

## INTRODUÇÃO

A tuberculose, apesar de curável, permanece como um dos desafios de saúde pública em escala mundial^1^. Em 2024, foi notificado pela Organização Mundial da Saúde (OMS) mais de 10,8 milhões de casos de tuberculose e 1,25 milhão de óbitos pela doença no mundo^2^.

O Brasil é um dos países com alta carga da doença, ocupando a 19ª posição mundial em número de casos, notificando 84.308 casos em 2024, o que representa uma incidência de 39,7 casos por 100 mil habitantes^3^; e um aumento de 21% no número de casos novos no período pós-pandemia de covid-19. Destaca-se que o estado de São Paulo foi responsável por aproximadamente 25,7% dos casos no Brasil em 2024, representando um cenário crítico para o controle da tuberculose^3^.

O conceito de recrudescimento da tuberculose, adotado neste estudo, é definido como o agravamento do cenário epidemiológico da doença após a fase aguda da pandemia de covid-19, caracterizado pelo aumento das interrupções de tratamento, mortes e aumento do número de casos, incluindo tuberculose multidroga-resistente^4^.

A pandemia de covid-19 impôs desafios significativos aos sistemas de saúde^5^, especialmente em regiões de alta carga de tuberculose. As medidas de distanciamento social, a sobrecarga dos serviços e a priorização no controle à pandemia comprometeram a continuidade do acompanhamento dos casos, afetando sobretudo populações vulnerabilizadas^5^.

As metas estabelecidas pela estratégia “*End* TB*”* de eliminar a tuberculose até 2050 (< 1 caso para cada 100.000 habitantes) foram profundamente impactadas pela pandemia da covid-19, o que vem impondo ao Sistema Único de Saúde (SUS), em seus diferentes níveis de gestão (federal, estadual e municipal), a implementação de medidas estratégicas no sentido de recuperação da situação epidemiológica e a eliminação da doença, conforme os compromissos assinados na 2ª Reunião de Alto Nível da Organização das Nações Unidas sobre Tuberculose^6^.

A necessidade de se alinhar as políticas, reestruturar os serviços de saúde em sinergia com a assistência social e outros setores estratégicos para recuperar o controle de declínio da incidência e da mortalidade, seguindo os Objetivos de Desenvolvimento Sustentável, notadamente no seu item 3.3^3^.

Todavia muitos estados não conseguiram se organizar quanto a essas diretrizes e os desafios, que já eram grandes na pré-pandemia, se intensificaram no pós-pandemia, com riscos de recrudescimento da tuberculose, pelos impactos desproporcionais em populações vulnerabilizadas^7^.

No estado de São Paulo, interrupções no diagnóstico afetaram especialmente comunidades periféricas, unidades prisionais e populações em situação de rua, onde barreiras de acesso ao diagnóstico, tratamento e acompanhamento agravam a subnotificação^8^. Essas lacunas possivelmente persistiram no período pós-pandemia, com recuperação desigual dos sistemas locais de saúde e vigilância em saúde, o que poderia levar ao recrudescimento da doença, carecendo de estudos com esse enfoque.

Estudos anteriores analisaram o impacto da covid-19 no contexto da tuberculose, mas não abordaram a dinâmica de maneira dual (tempo e espaço) para o pós-pandemia^7,8^. Este estudo, portanto, apresenta uma contribuição original ao investigar o recrudescimento da tuberculose no período pós-pandêmico.

A abordagem permite correlacionar fatores locais, proporcionando evidências mais abrangentes sobre a dinâmica multidimensional da doença, distinguindo-se das análises tradicionais baseadas em dados agregados. Assim, o objetivo do estudo foi analisar o recrudescimento da tuberculose no estado de São Paulo no período pós-pandêmico da covid-19, tendências temporais e *clusters* espaciais da doença.

## MÉTODOS

### Desenho do Estudo

Trata-se de um estudo ecológico exploratório de séries temporais e análise espacial^9^. Utilizou-se como unidade de análise os 645 municípios do estado de São Paulo.

### População do Estudo

No presente trabalho, foram analisados todos os casos notificados de tuberculose no estado de São Paulo, abrangendo todas as faixas etárias e formas clínicas da doença, no período de janeiro de 2015 a dezembro de 2023. Para avaliar o recrudescimento, o período analisado foi dividido em três fases distintas: (i) Pré-pandêmica (janeiro de 2015 a janeiro de 2020); (ii) Pandêmica (fevereiro de 2020 a abril de 2022); e (iii) Pós-pandêmica (maio de 2022 a dezembro de 2023).

O início do período pandêmico foi definido a partir de fevereiro de 2020, data do primeiro caso notificado de covid-19 no Brasil e no estado de São Paulo^10^. O término desse período foi estabelecido em abril de 2022, quando houve a declaração oficial do fim da Emergência de Saúde Pública de Importância Nacional (ESPIN) relacionada à covid-19^11^.

Os dados de tuberculose foram obtidos do Sistema de Controle de Pacientes com Tuberculose (TBWeb), disponibilizados pela Secretaria de Estado da Saúde de São Paulo e pela Divisão de Tuberculose do Centro de Vigilância Epidemiológica “Prof. Alexandre Vranjac” (CVE), do estado de São Paulo. As estimativas populacionais utilizadas para análise foram obtidas do Instituto Brasileiro de Geografia e Estatística (IBGE)^12^.

### Local de Estudo

O estado de São Paulo está localizado na região sudeste do Brasil e é o mais populoso do país, com mais de 44 milhões de habitantes em 2022, o que representa aproximadamente 22% da população brasileira. Além disso, os 645 municípios do estado são organizados administrativamente em 63 microrregiões e 17 Departamentos Regionais de Saúde^12^.

### Classificação das Variáveis

As variáveis utilizadas no estudo foram organizadas de acordo com o tipo de análise realizada: descritiva, temporal e espacial.

### Variáveis Utilizadas para as Análises Descritivas

As variáveis foram obtidas a partir dos dados da Ficha de Notificação de Tuberculose do Sistema TBWeb, a saber: (i) sexo, (ii) faixa etária em anos (0–14,15–29,30–39, 40–49, 50–59 e ≥ 60 anos), (iii) raça/cor (branca, parda, negra, amarela e indígena), (iv) escolaridade em anos de estudo (sem escolaridade, 1–3, 4–7, 8–11, 12–14, ≥ 15 anos), (v) tipo de entrada, (vi) diagnóstico (teste rápido molecular, histopatologia, baciloscopia, cultura, teste HIV, sensibilidade, raio-x), (vii) forma clínica, (viii) encerramento de caso, (ix) terapia antirretroviral, (x) comorbidade (aids/HIV, diabetes, drogadição, etilismo, tabagismo), (xi) população em situação de rua e população privada de liberdade.

### Variáveis Utilizadas para Análises Temporais

Para esta fase, os casos notificados e os óbitos de tuberculose foram agregados de acordo com o porte populacional dos municípios e critérios da Secretaria de Desenvolvimento Social do Estado de São Paulo (SEDS-SP)^13^, a saber: (a) metrópole: população superior a 900.001 habitantes; (b) grande porte: população entre 100.001 a 900.000 habitantes; (c) médio porte: população entre 50.001 e 100.000 habitantes; (d) pequeno porte I: população até 20.000 habitantes; e (e) pequeno porte II: população entre 20.001 e 50.000 habitantes. A categorização permitiu a análise das tendências epidemiológicas, conforme o perfil demográfico dos municípios.

A taxa de incidência de tuberculose foi calculada conforme a fórmula:


$$\frac{\text{ número de casos mês }}{\text{ população }}*100.000\text{ habitantes }$$

A taxa de mortalidade de tuberculose foi calculada com a fórmula:


$$\frac{\text{ número de óbitos mês }}{\text{ população }}*100.000\text{ habitantes }$$

Além disso, calculou-se:

A proporção de óbitos de tuberculose:


$$\frac{\text{ número óbitos mês }}{\text{ casos de tuberculose mês }}*100$$

A proporção de cura de tuberculose:


$$\frac{\text{ número de casos cura mês }}{\text{ casos de tuberculose mês }}*100$$

A proporção de interrupção de tratamento de tuberculose:


$$\frac{\text{ número de casos de interrupção do tratamento mês }}{\text{ casos de tuberculose mês }}*100$$

### Variáveis Utilizadas para as Análises Espaciais (Clusters Espaciais)

Para identificar áreas críticas de recrudescimento da tuberculose e padrões espaciais, foram calculadas, por município, as taxas de incidência e mortalidade de tuberculose, bem como as proporções de cura, óbito e interrupção de tratamento, considerando quatro períodos: (i) total do estudo; (ii) pré-pandêmico; (iii) pandêmico e (iv) pós-pandêmico.

As taxas de incidência e mortalidade por tuberculose foram calculadas, conforme as fórmulas abaixo:

Taxa de incidência de tuberculose:


$$\frac{\text{ número de casos no município/meses }}{\text{ população no município }}*100.000\text{ habitantes }$$

Taxa de mortalidade de tuberculose:


$$\frac{\text{ número de óbitos no município/meses }}{\text{ população no município }}*100.000\text{ habitantes }$$

### Análise Descritiva

A análise das variáveis sociodemográficas e clínico-epidemiológicas foram realizadas por meio de estatística descritiva dos parâmetros quantitativos, sendo calculadas as frequências absolutas e relativas para as variáveis.

Realizaram-se as análises descritivas utilizando a linguagem R^®^ (versão 4.0.3) por meio da interface RStudio^®^ (2024.12.1), com o pacote estatístico *summarytools.*

### Tendência Temporal dos Casos de Tuberculose

Aplicou-se a decomposição *Seasonal-Trend Decomposition using Loess* (STL)*,* método robusto que decompõe séries temporais em três componentes mediante regressão localmente ponderada: tendência, sazonalidade e resíduo^14^. O estudo focou exclusivamente na análise da componente de tendência, permitindo caracterizar o comportamento temporal das variáveis de morbimortalidade por tuberculose.

Para avaliar o recrudescimento, definiu-se dois marcos temporais no período pandêmico, tomando por base evidências epidemiológicas:

- Marco A (agosto/2021): período em que 50% da população brasileira recebeu ao menos uma dose da vacina contra covid-19, 22% completaram o esquema vacinal, e observou-se redução de 40% na mortalidade por covid-19^15^;
- Marco B (abril/2022): corresponde ao decreto de encerramento da Emergência em Saúde Pública de Importância Nacional (ESPIN) no Brasil^11^.

### Série Temporal Interrompida

Utilizou-se a técnica de série temporal interrompida (STI), conduzida por meio de modelos autorregressivos integrados de médias móveis (ARIMA), de acordo com o método de Box-Jenkins^16^, com o objetivo de avaliar o impacto da pandemia de covid-19 sobre os desfechos selecionados. A abordagem adotada seguiu a do modelo proposto por Schaffer et al.^17^ (2021), que recomenda o uso de modelos ARIMA para a avaliação de intervenções em larga escala em saúde pública, com base em séries temporais.

Inicialmente, foi verificada a estacionariedade das séries por meio de inspeção visual, testes estatísticos e análise das funções de autocorrelação (ACF) e autocorrelação parcial (PACF). Quando necessário, aplicaram-se diferenciações sazonais e não sazonais para estabilizar a média e remover tendências ou padrões sazonais.

A identificação da ordem dos componentes autorregressivos (p), de diferenciação (d), e de média móvel (q), bem como dos componentes sazonais (P, D, Q), foi realizada com base na inspeção dos gráficos de ACF e PACF e na minimização dos critérios de informação (*Akaike Information Criterion* – AIC)^16^. Fez-se o ajuste dos modelos de forma iterativa até que os resíduos apresentassem comportamento de ruído branco, verificado com os testes de Ljung-Box, Box-Pierce, Turning Point, T-test e Kolmogorov-Smirnov.

Para estimar o impacto da pandemia, foram incluídas variáveis indicadoras de intervenção representando mudanças abruptas (*step*) e mudanças na inclinação da tendência (*ramp* ou *slope*), com março de 2020 definido como ponto inicial da intervenção. A significância estatística dos coeficientes estimados foi avaliada por meio de intervalos de confiança de 95% (IC95%).

### Associação Espacial da Tuberculose

Para avaliar a autocorrelação espacial, utilizou-se uma abordagem exploratória baseada nos testes de Moran’s I e Getis-Ord Gi*. O índice global de Moran (Moran’s I) foi aplicado para verificar a presença, força e direção da autocorrelação espacial entre os municípios do estado, com base em uma matriz de vizinhança do tipo *Queen* de ordem 1. Esse índice varia de -1 a +1, sendo que valores positivos indicam associação espacial direta (agrupamento de valores semelhantes), enquanto valores negativos indicam associação inversa. Enquanto que valores próximos de zero sugerem aleatoriedade espacial. Considerou-se significância estatística para p < 0,05^18^. A análise do Moran’s I foi realizada no software GeoDa^®^, versão 1.22.0.14^19^.

Em escala local, foi utilizado o teste de Getis-Ord Gi* para detectar agrupamentos espaciais significativos e identificar áreas quentes (*hotspots*) e frias (*coldspots*). O valor de Gi*(d) é expresso por uma pontuação Z, que pode variar de -3 a +3, indicando, respectivamente, agrupamentos significativos de baixos e altos valores. Quanto maior o valor da pontuação Z, mais forte é o agrupamento de altas taxas (*hotspots*), enquanto valores negativos indicam agrupamentos de baixas taxas *(coldspots*). Valores próximos de zero indicam ausência de agrupamento significativo^20^. Nas análises do Gi*, considerou-se três níveis de significância estatística: 90%, 95% e 99%, o que permitiu uma classificação mais refinada da intensidade dos agrupamentos espaciais.

A malha territorial dos municípios do estado foi obtida do acervo do IBGE^12^, e os mapas coropléticos foram elaborados no software ArcGIS^®^ versão 10.5.

### Aspectos Éticos

O estudo foi aprovado pelo Comitê de Ética em Pesquisa da Escola de Enfermagem de Ribeirão Preto da Universidade de São Paulo (EERP/USP), sob o número do Certificado de Apresentação de Apreciação Ética (CAAE) 77580424.5.0000.5393, em conformidade com as Resoluções nº 466/2012 e nº 510/2016 do Conselho Nacional de Saúde.

## RESULTADOS

### Análise Descritiva

A Tabela 1 traz a descrição das variáveis sociodemográficas e clínico-epidemiológicas dos casos notificados de tuberculose. Entre 2015 e 2023, foram notificados 196.950 casos de tuberculose no estado de São Paulo. Desse total, 55,9% (n = 110.051) ocorreram no período pré-pandêmico, 23,3% (n = 45.926) durante a pandemia e 20,8% (n = 40.973) no pós-pandêmico, evidenciando-se queda nas notificações recentes. O perfil dos casos manteve predominância masculina (n = 142.767; 72,5%), com idade média de 39 anos.

**Tabela 1:** Características sociodemográficas e clínicas de pessoas diagnosticadas com tuberculose, no estado de São Paulo, no período de 2015–2023.

Variáveis	Acumulado (2015–2023)	Pré-pandêmico (01/2015–01/2020)	Pandêmico (02/2020–04/2022)	Pós-pandêmico (05/2022–12/2023)
	n (%)	n (%)	n (%)	n (%)
Total	196.950 (100)	110.051 (55,9)	45.926 (23,3)	40.973 (20,8)
Sexo				
Masculino	142.767 (72,5)	80.358 (73,0)	32.975 (71,8)	29.434 (71,8)
Feminino	54.183 (27,5)	29.693 (27,0)	12.951 (28,2)	11.539 (28,2)
Faixa etária (anos)				
0–14	4.500 (2,3)	2.563 (2,3)	916 (2,0)	1.021 (2,5)
15–29	58.136 (29,5)	33.936 (30,8)	13.164 (28,7)	11.036 (27,0)
30–39	47.206 (24,0)	26.479 (24,1)	11.001 (24,0)	9.726 (23,7)
40–49	35.648 (18,1)	19.130 (17,4)	8.561 (18,6)	7.957 (19,4)
50–59	26.311 (13,4)	14.579 (13,2)	6.191 (13,5)	5.541(13,5)
≥ 60	24.852 (12,6)	13.184 (12,0)	6.032 (13,1)	5.636 (13,8)
Sem informação	297 (0,1)	180 (0,2)	61 (0,1)	56 (0,1)
Raça/Cor				
Branca	71.171 (36,1)	44.017 (40,0)	16.540 (36,0)	10.614 (25,9)
Preta	20.364 (10,3)	11.773 (10,7)	5.172 (11,3)	3.419 (8,4)
Amarela	1.344 (0,7)	787 (0,72)	345 (0,8)	212 (0,5)
Parda	67.897 (34,5)	39.298 (35,7)	17.124 (37,3)	11.475 (28,0)
Indígena	404 (0,2)	282 (0,3)	69 (0,2)	53 (0,1)
Sem informação	35.770 (18,2)	13.894 (12,6)	6.676 (14,5)	15.200 (37,1)
Nível Escolaridade (anos)				
Sem escolaridade	4.653 (2,4)	2.813 (2,6)	902 (2,0)	938 (2,3)
1 a 3	13.137 (6,7)	7.888 (7,2)	2.902 (6,3)	2.347 (5,7)
4 a 7	50.857 (25,8)	30.143 (27,4)	11.155 (24,3)	9.559 (23,3)
8 a 11	57.866 (29,4)	32.473 (29,5)	13.595 (29,6)	11.798 (28,8)
12 a 14	13.030 (6,6)	6.845 (6,2)	3.261 (7,1)	2.924 (7,1)
≥ 15	5.297 (2,7)	2.975 (2,7)	1.268 (2,8)	1.054 (2,6)
Sem informação	52.110 (26,5)	26.914 (24,5)	12.843 (28)	12.353 (30,1)
Tipo de entrada				
Caso novo	159.683 (81,0)	90.463 (82,2)	36.895 (80,3)	32.325 (78,9)
Recidiva	19.251 (9,8)	10.847 (9,9)	4.512 (9,8)	3.892 (9,5)
Retratamento após interrupção	15.376 (7,9)	7.575 (6,9)	3.708 (8,1)	4.093 (10)
Retratamento após falência/resistência	1.445 (0,7)	796 (0,7)	353 (0,8)	296 (0,7)
Retratamento após mudança esquema	1.195 (0,6)	370 (0,3)	458 (1,0)	367 (0,9)
Histopatologia				
BAAR positivo	3.638 (1,9)	1.990 (1,8)	930 (2,0)	718 (1,8)
Sugestivo de tuberculose	8.305 (4,2)	5.017 (4,6)	1.907 (4,2)	1.381 (3,4)
Não realizado	141.752 (72,0)	78.827 (71,6)	32.343 (70,4)	30.582 (74,6)
Sem informação	43.255 (22,0)	24.217 (22,0)	10.746 (23,4)	8.292 (20,2)
Baciloscopia				
Positiva	92.581 (47,0)	54.098 (49,2)	20.779 (45,2)	17.704 (43,2)
Negativa	40.337 (20,5)	23.990 (21,8)	8.615 (18,8)	7.732 (18,9)
Em andamento	760 (0,4)	296 (0,3)	135 (0,3)	329 (0,8)
Não realizada	61.423 (31,2)	30.819 (28)	15.903 (34,6)	14.701 (35,9)
Sem informação	1.849 (0,9)	848 (0,8)	494 (1,1)	507 (1,2)
Cultura				
Positivo	67.676 (34,4)	39.527 (35,9)	15.015 (32,7)	13.134 (32,1)
Negativo	23.873 (12,1)	13.773 (12,5)	5.598 (12,2)	4.502 (11,0)
Em andamento	2.464 (1,3)	949 (0,9)	342.0 (0,7)	1.173 (2,9)
Não realizado	97.287 (49,4)	52.521 (47,7)	23.758 (51,7)	21.008 (51,3)
Sem informação	5.650 (2,9)	3.281 (3,0)	1.213 (2,6)	1.156 (2,8)
Teste do Vírus da Imunodeficiência Humana (HIV)				
Positivo	18.018 (9,2)	10.405 (9,5)	3.875 (8,4)	3.738 (9,1)
Negativo	160.738 (81,6)	89.410 (81,2)	38.049 (82,9)	33.279 (81,2)
Não realizado	12.550 (6,4)	7.443 (6,8)	2.727 (5,9)	2.380 (5,8)
Em andamento	983 (0,5)	432.0 (0,4)	179 (0,4)	372 (0,9)
Sem informação	4.661 (2,4)	2.361 (2,2)	1.096 (2,4)	1.204 (2,9)
Teste de sensibilidade				
Sim	48.681 (24,7)	24.603 (22,4)	12.693 (27,6)	11.385 (27,8)
Não	113.269 (57,5)	56.452 (51,3)	29.856 (65,0)	26.961 (65,8)
Sem informação	35.000 (17,8)	28.996 (26,4)	3.377 (7,4)	2.627 (6,4)
Exame de Raio-X				
Normal	10.199 (5,2)	5.994 (5,5)	2.283 (5,0)	1.922 (4,7)
Suspeita de tuberculose	95.607 (48,5)	55.332 (50,3)	21.705 (47,3)	18.570 (45,3)
Suspeita de tuberculose, com cavidade	25.633 (13,0)	14.539 (13,2)	5.977 (13,0)	5.117 (12,5)
Outra patologia	2.280 (1,2)	1.335 (1,2)	446 (1,0)	499 (1,2)
Não realizado	47.213 (24,0)	24.259 (22,0)	11.628 (25,3)	11.326 (27,6)
Sem informação	16.018 (8,1)	8.592 (7,8)	3.887 (8,5)	3.539 (8,6)
Forma clínica				
Pulmonar	163.973 (83,3)	91.498 (83,1)	38.126 (83,0)	34.349 (83,8)
Extrapulmonar	26.388 (13,4)	14.989 (13,6)	6.196 (13,5)	5.203 (12,7)
Pulmonar + extrapulmonar	5.991 (3,0)	3.256 (3,0)	1.449 (3,2)	1.286 (3,1)
Disseminada	588 (0,3)	298 (0,3)	155 (0,3)	135 (0,3)
Sem informação	10 (0)	10 (0)	0 (0)	0 (0)
Encerramento				
Interrupção de tratamento	29.634 (15,1)	14.228 (12,9)	7.763 (16,9)	7.643 (18,7)
Cura	142.387 (72,3)	84.720 (77,0)	32.486 (70,7)	25.181 (61,5)
Falência/resistência	1.475 (0,8)	804 (0,7)	358 (0,8)	313 (0,8)
Mudança de esquema	1.426 (0,7)	465 (0,4)	532 (1,2)	429 (1,1)
Óbito	15.760 (8,0)	8.292 (7,5)	3.952 (8,6)	3.516 (8,6)
Transferência	1.718 (0,9)	882 (0,8)	444 (1,0)	392 (1,0)
Sem informação	4.550 (2,3)	660 (0,6)	391 (0,9)	3.499 (8,5)
Teste molecular rápido de tuberculose (TRM-TB)				
Detectável, sensível a rifampicina	68.948 (35,0)	32.635 (29,7)	18.270 (39,8)	18.043 (44,0)
Detectável, resistente a rifampicina	2.043 (1,0)	1.038 (0,9)	509 (1,1)	496 (1,2)
Detectável, rifampicina indeterminado	1.367 (0,7)	247 (0,2)	502 (1,1)	618 (1,5)
Não detectável	13.162 (6,7)	6.654 (6,1)	3.145 (6,9)	3.363 (8,2)
Inconclusivo	513 (0,3)	269 (0,2)	118 (0,3)	126 (0,3)
Não realizado	80.146 (40,7)	48.520 (44,1)	17.580 (38,3)	14.046 (34,3)
Sem informação	30.771 (15,6)	20.688 (18,8)	5.802 (12,6)	4.281 (10,5)
Terapia antirretroviral				
Sim	8.579 (4,4)	4.164 (3,8)	2.166 (4,7)	2.249 (5,5)
Não	2.236 (1,1)	1.149 (1,0)	571 (1,2)	516 (1,3)
Sem informação	186.135 (94,5)	104.738 (95,2)	43.189 (94,0)	38.208 (93,3)
População em situação de rua				
Sim	10.019 (5,1)	5.169 (4,7)	2.417 (5,3)	2.433 (5,9)
Não	186.931 (94,9)	104.882 (95,3)	43.509 (94,7)	38.540 (94,1)
População privada de liberdade				
Sim	23.904 (12,1)	15.800 (14,4)	4.734 (10,3)	3.370 (8,2)
Não	173.046 (87,9)	94.251 (85,6)	41.192 (89,7)	37.603 (91,8)
Imigrantes				
Sim	1.395 (0,7)	872 (0,8)	337 (0,7)	186 (0,5)
Não	119.649 (60,8)	76.401 (69,4)	27.635 (60,2)	15.613 (38,1)
Sem informação	75.906 (38,5)	32.778 (29,8)	17.954 (39,1)	25.174 (61,4)
Aids				
Sim	16.749 (8,5)	9.714 (8,8)	3.599 (7,8)	3.436 (8,4)
Não	180.201 (91,5)	100.337 (91,2)	42.327 (92,2)	37.537 (91,6)
Diabetes				
Sim	13.421 (6,8)	6.953 (6,3)	3.575 (7,8)	2.893 (7,1)
Não	183.529 (93,2)	103.098 (93,7)	42.351 (92,2)	38.080 (92,9)
Etilismo				
Sim	39.259 (19,9)	20.861 (19,0)	9.354 (20,4)	9.044 (22,1)
Não	157.691 (80,1)	89.190 (81,0)	36.572 (79,4)	31.929 (77,9)
Tabagismo				
Sim	51.802 (26,3)	27.335 (24,8)	12.478 (27,2)	11.989 (29,3)
Não	145.148 (73,7)	82.716 (75,2)	33.448 (72,8)	28.984 (70,7)
Doença mental				
Sim	3.024 (1,5)	1.639 (1,5)	727 (1,6)	658 (1,6)
Não	193.926 (98,5)	108.412 (98,5)	45.199 (98,4)	40.315 (98,4)
Drogadição				
Sim	39.052 (19,8)	20.745 (18,9)	9.118 (19,9)	9.189 (22,4)
Não	157.898 (80,2)	89.306 (81,2)	36.808 (80,1)	31.784 (77,6)
Outra doença imunológica				
Sim	2.359 (1,2)	1.316 (1,2)	556 (1,2)	487 (1,2)
Não	194.591 (98,8)	108.735 (45,4)	45.370 (98,8)	40.486 (98,8)

Observou-se uma mudança expressiva no perfil racial: enquanto a categoria branca era predominante antes da pandemia (n = 44.017; 40,0%), houve inversão nos períodos pandêmico (n = 17.124; 37,3%) e pós-pandêmico (n = 11.475; 28%), com aumento relativo de casos entre pessoas pardas. Destaca-se, ainda, o crescimento acentuado de dados ausentes para raça/cor e escolaridade no pós-pandêmico, o que pode indicar fragilidades na vigilância e dificultar análises mais detalhadas sobre desigualdades.

A maioria dos casos foi classificada como nova ocorrência, porém, a proporção caiu de 82,2% (n = 90.463) no pré-pandêmico para 78,9% (n = 32.325) no pós-pandêmico, enquanto os casos de retratamento após interrupção aumentaram de 6,9% (n = 7.575) para 10,0% (n = 4.093), sugerindo reflexo da pandemia na continuidade do cuidado. A forma pulmonar permaneceu predominante (n = 163.973; 83,3%).

Os desfechos revelam agravamento do cenário: a cura despencou de 77,0% (n = 84.720) no pré-pandêmico para 61,5% (n = 25.181) no pós-pandêmico, ao passo que a interrupção do tratamento saltou de 12,9% (n = 14.228) para 18,7% (n = 7.643). Esse quadro sinaliza ruptura na adesão e maior risco de transmissão e resistência.

Entre as comorbidades, a prevalência de pessoas vivendo com HIV manteve-se estável em todo o período, mas houve aumento nos casos associados ao diabetes durante a pandemia. Fatores sociais de risco, como etilismo, tabagismo e uso de drogas, apresentaram crescimento consistente no pós-pandêmico, indicando agravamento das vulnerabilidades sociais.

No recorte das populações vulneráveis, chama a atenção a expressiva redução na notificação de casos entre pessoas privadas de liberdade (de 14,4%; [n = 15.800] para 8,2% [n = 3.370]), enquanto os casos entre pessoas em situação de rua aumentaram levemente de 4,7% (n = 5.169) para 5,9% (n = 2.433), sugerindo possíveis falhas no rastreamento e acesso ao diagnóstico nessas populações.

### Tendência Temporal e Série Temporal Interrompida

A Figura 1 traz as tendências temporais da incidência, mortalidades e desfechos de tratamento da tuberculose.

**Figura 1:** Tendências das taxas de incidência e mortalidade e desfechos de tratamento da tuberculose nos municípios do estado de São Paulo, de acordo com categorias de porte populacional, no período de 2015 a 2023.

A análise dos comportamentos da incidência e mortalidade permite notar alterações importantes a partir de 2020. Entre o início da pandemia e 2021, a notificação dos casos apresentam redução entre todos os portes de municípios e principalmente nas metrópoles. A partir de 07/2021, a notificação dos casos passa a crescer de forma gradual, atingindo máximas históricas no final de 2023 no estado e entre municípios de grande porte e metrópoles. Os municípios de menor porte (médio e pequeno I e II) apresentaram tendências semelhantes, contudo com variações mais discretas ao longo do tempo.

De forma semelhante, houve aumento das taxas de mortalidade após 2021, atingindo maiores valores mensais das séries em 2022 e iniciando uma redução ao final de 2023.

Entre os desfechos de tratamento, destaca-se a redução acentuada na proporção dos casos encerrados como cura, desde o início de 2020 até o final de 2023 de maneira consistente. Inversamente, observou-se o aumento das proporções de interrupção de tratamento, mas que já apresentavam indicativos de aumento antes do início da pandemia.

A Tabela 2 evidencia os resultados da aplicação da análise de STI, com os respectivos modelos ARIMA ajustados e as estimativas dos efeitos da intervenção (*step* e *slope*). Adicionalmente, são apresentados os resultados do teste de Ljung-Box, utilizado para verificar a presença de autocorrelação nos resíduos, e o *Mean Absolute Percentage Error* (MAPE), que avalia a acurácia dos modelos com base na média dos erros percentuais absolutos entre os valores previstos e os valores observados.

**Tabela 2:** Modelagem de séries temporais interrompidas ARIMA para avaliação das tendências da incidência, mortalidade e desfechos de tratamento da tuberculose no estado de São Paulo.

Indicador/porte municipal	Modelo ARIMA	Step (IC95%)	Slope (IC95%)	MAPE	Ljung-Box (valor-p)
Incidência					
Estado de São Paulo	(0,0,2)(1,0,0)	-0,69 (-0,94 a -0,44)	0,034 (0,025 a 0,043)	5,62	27,44 (0,28)
Grande porte	(0,0,0)(1,0,0)	-0,54 (-0,76 a -0,33)	0,030 (0,022 a 0,037)	6,69	24,51 (0,43)
Médio porte	(0,0,0)(1,0,0)	-0,40 (-0,67 a -0,12)	0,025 (0,016 a 0,035)	12,23	17,02 (0,85)
Metrópole	(0,0,5)	-0,91 (-1,31 a -0,51)	0,052 (0,038 a 0,065)	6,83	20,11 (0,69)
Pequeno porte I	(2,0,0)(1,0,1)	-0,70 (-1,36 a -0,05)	0,006 (-0,016 a 0,029)	13,58	33,57 (0,09)
Pequeno porte II	(0,1,1)(2,0,0)	-0,22 (-0,69 a 0,25)	0,023 (-0,025 a 0,070)	10,59	27,45 (0,28)
Mortalidade					
Estado de São Paulo	(2,0,2)	-0,0098 (-0,0371 a 0,0174)	0,0026 (0,0016 a 0,0035)	8,44	15,10 (0,92)
Grande porte	(1,0,1)	0,0023 (-0,0415 a 0,0461)	0,0021 (0,0007 a 0,0035)	12,51	16,52 (0,87)
Médio porte	(2,1,3)	0,0294 (-0.0088 a 0.0676)	0.0005 (-0.0008 a 0,0018)	32,91	13,65 (0,95)
Metrópole	(0,1,1)	-0,0206 (-0,0688 a 0,0276)	0,0039 (0,0022 a 0,0056)	11,44	28,13 (0,25)
Pequeno porte I	(2,1,0)	-0,0409 (-0,1653 a 0,0836)	0,0000 (-0,0119 a 0,0119)	∞	32,47 (0,11)
Pequeno porte II	(0,1,1)	0,5725 (-1,8004 a 2,9455)	-0,0016 (-0,0504 a 0,0472)	50,37	19,86 (0,70)
Cura					
Estado de São Paulo	(1,1,0)	1,946 (-1,498 a 5,389)	-0,672 (-1,099 a -0,246)	1,95	19,74 (0,71)
Grande porte	(1,0,4)	1,961 (-2,828 a 6,749)	-1,036 (-1,617 a -0,455)	2,67	12,80 (0,97)
Médio porte	(0,0,3)	0,040 (-5,712 a 5,792)	-0,444 (-0,642 a -0,245)	5,32	13,16 (0,96)
Metrópole	(2,0,0)	2,480 (-1,815 a 6,774)	-0,573 (-0,779 a -0,368)	2,80	13,04 (0,97)
Pequeno porte I	(1,1,1)(2,0,0)	1,726 (-1,424 a 4,875)	-0,294 (-0,401 a -0,187)	3,67	18,41 (0,78)
Pequeno porte II	(1,0,1)	0,030 (-5,376 a 5,436)	-0,416 (-0,625 a -0,206)	4,46	16,24 (0,88)
Interrupção do tratamento					
Estado de São Paulo	(1,1,1)	0,507 (-1,134 a 2,148)	0,063 (-0,024 a 0,149)	5,91	14,24 (0,94)
Grande porte	(1,1,2)	1,261 (-0,451 a 2,973)	0,042 (-0,050 a 0,134)	8,16	15,99 (0,89)
Médio porte	(0,1,1)	2,784 (0,817 a 4,750)	0,040 (-0,027 a 0,107)	18,66	24,09 (0,46)
Metrópole	(0,0,1)	2,057 (0,756 a 3,358)	0,106 (0,061 a 0,150)	7,31	15,32 (0,91)
Pequeno porte I	(0,1,3)(0,0,1)	-0,940 (-4,564 a 2,684)	0,032 (-0,109 a 0,173)	∞	31,55 (0,14)
Pequeno porte II	(0,1,1)	0,876 (-2,377 a 4,130)	0,081 (-0,026 a 0,189)	30,15	16,61 (0,86)
Óbitos					
Estado de São Paulo	(0,1,1)	1,339 (0,852 a 1,826)	-0,010 (-0,027 a 0,006)	7,55	22,48 (0,55)
Grande porte	(1,1,2)	1,441 (0,838 a 2,044)	-0,007 (-0,028 a 0,014)	9,85	20,33 (0,68)
Médio porte	(1,0,1)	1,979 (1,552 a 2,406)	-0,030 (-0,047 a -0,013)	31,99	25,72 (0,37)
Metrópole	(2,1,2)	1,695 (0,474 a 2,916)	-0,048 (-0,130 a 0,035)	10,12	17,32 (0,83)
Pequeno porte I	(0,1,1)	0,613 (-0,748 a 1,973)	0,026 (-0,020 a 0,073)	∞	19,10 (0,75)
Pequeno porte I	(0,1,1)	0,573 (-1,800 a 2,945)	-0,002 (-0,050 a 0,047)	50,37	22,95 (0,52)

IC95%: intervalo de confiança de 95%; MAPE: *mean absolute percentage error*; ARIMA: modelos autorregressivos integrados de médias móveis; ∞: infinito.

A aplicação da técnica revelou que a pandemia da covid-19 teve impactos estatisticamente significativos em diferentes indicadores da tuberculose no estado de São Paulo, variando conforme o porte municipal.

Para a incidência da tuberculose, identificou-se redução imediata entre todos os recortes populacionais, com exceção nos municípios de pequeno porte II. As metrópoles apresentaram a maior redução no *step* em 0,91 caso por 100 mil habitantes (IC95% -1,31 a -0,51), possivelmente refletindo subnotificação de casos nos primeiros meses da pandemia. Os municípios de maior porte (Metrópole, grande e médio) demonstraram tendências de aumentos após a pandemia, chegando a 0,052 caso por 100 mil habitantes/mês nas metrópoles.

Em relação à mortalidade por tuberculose, observou-se uma tendência crescente ao longo do tempo no estado após o início da pandemia (*slope*: 0,0026; IC95% 0,0016 a 0,0035), indicando um agravamento progressivo do indicador. Esse mesmo padrão foi identificado nos municípios de grande porte e metrópoles.

Para a proporção de cura, não foram detectadas alterações imediatas na série com início da pandemia. Por outro lado, todas as categorias de portes apresentaram tendências de quedas com destaque para municípios de grande porte com redução de 1,03% por mês (IC95% -1,62 a -0,46).

No indicador de interrupção do tratamento, houve aumento imediato em municípios de médio porte (*step*: 2,783; IC95% 0,8171 a 4,750) e em regiões metropolitanas (*step*: 2,056; IC95% 0,7559 a 3,357). Ainda, os modelos apontaram uma tendência crescente ao longo do tempo nas metrópoles (*slope*: 0,1058; IC95% 0,0614 a 0,1502).

Os óbitos por tuberculose também foram significativamente afetados: observou-se aumento imediato no estado (*step*: 1,339; IC95% 0,852 a 1,826), em municípios de grande porte (*step*: 1,441; IC95% 0,838 a 2,044), de médio porte (*step*: 1,979; IC95% 1,552 a 2,406), em regiões metropolitanas (*step*: 1,695; IC95% 0,474 a 2,916).

Os modelos ajustados para todas as séries foram aqueles que apresentaram melhor capacidade preditiva e menor valor de AIC em comparação com as demais opções testadas. Além disso, todos os modelos selecionados tiveram resíduos não autocorrelacionados e com distribuição normal, atendendo aos pressupostos fundamentais da modelagem. Dessa forma, os modelos ajustados demonstraram validade para fins preditivos e analíticos.

### Associação Espacial da Incidência e Mortalidade por Tuberculose

A distribuição espacial da incidência por tuberculose no estado de São Paulo demonstra que a distribuição é heterogênea, e mantém padrões de distribuição semelhantes ao longo dos períodos estudados, com uma concentração de municípios com taxas mais elevadas na região de Presidente Prudente, Araçatuba, Bauru e Baixada Santista. Destaca-se que, ao comparar o período pandêmico com o pós-pandemia, observa-se um aumento no número de municípios com incidência de 110 casos para cada 100.000 habitantes (Figura 2). As taxas de mortalidade por tuberculose apresentaram um aumento amplamente distribuído em todo o estado após a pandemia. Paralelamente, os municípios sem notificação de óbitos aumentaram (Figura 2).

**Figura 2:** Distribuição espacial das taxas de incidência e mortalidade da tuberculose, São Paulo, Brasil, 2015–2023.

Ao longo do período analisado, observou-se a presença de autocorrelação espacial positiva, com variações na intensidade conforme a fase da pandemia. Para a taxa de incidência, a autocorrelação global medida pelo índice global de Moran apresentou valor moderado no período acumulado (2015–2023), com Moran’s I = 0,15 (p < 0,01). No período pré-pandêmico, o valor foi ligeiramente maior, Moran’s I = 0,19 (p < 0,01), indicando um padrão mais acentuado de agrupamento espacial.

Durante o período pandêmico, a autocorrelação diminuiu consideravelmente, com Moran’s I = 0,05 (p = 0,01), sugerindo um enfraquecimento da estrutura espacial. No pós-pandêmico, houve discreto aumento, com Moran’s I = 0,09 (p < 0,01).

Em relação à mortalidade, também foi identificada a autocorrelação espacial positiva ao longo de todos os períodos. No acumulado, o valor foi de 0,13 (p < 0,01). No período pré-pandêmico, a autocorrelação foi semelhante, com Moran’s I = 0,12 (p < 0,01). Durante a pandemia, a intensidade do agrupamento espacial foi mais baixa, com Moran’s I = 0,051 (p = 0,02), e voltou a crescer levemente no período pós-pandêmico, atingindo 0,08 (p < 0,01).

Os resultados da análise do Gi* (Figura 3) mostram a formação dos *hotspots* de alta incidência da tuberculose no estado de São Paulo, evidenciando a persistência de elevada carga da doença na região de Presidente Prudente, dispersando aos demais municípios com altas taxas ao longo do tempo.

**Figura 3:** Municípios do estado de São Paulo que apresentam associação espacial local Getis-Ord Gi* para as taxas de incidência e mortalidade por tuberculose, Brasil, 2015–2023.

Na região de Marília e Bauru, observou-se uma mudança no padrão espacial: enquanto os *hotspots* estavam concentrados nos municípios mais ao norte no período pré-pandêmico, após a pandemia a concentração de casos passou a ocorrer nos municípios do sul da região, sugerindo uma dispersão geográfica da transmissão. Ainda no período pós-pandêmico, destaca-se o surgimento de um novo *hotspot* na região de Registro, o que indica possível recrudescimento local da transmissão da doença.

Em relação aos *hotspots* de mortalidade por tuberculose, notou-se que as regiões de Taubaté, Registro, Baixada Santista e Grande São Paulo apresentaram elevada taxa de mortalidade em todos os períodos analisados. As regiões de Sorocaba e Campinas mostraram aglomerados no período pré-pandêmico, os quais se dispersaram pelos municípios e tornaram-se mais evidentes a partir da pandemia, sugerindo agravamento do cenário epidemiológico (Figura 3).

## DISCUSSÃO

O estudo evidenciou que o recrudescimento pós-pandêmico da tuberculose em São Paulo é caracterizado pela elevação na incidência, interrupção de tratamento, aumento da mortalidade e redução nas taxas de cura. Identificou-se a conformação de *clusters* em áreas com baixa cobertura da atenção primaria à saúde, indicando que fatores contextuais locais e a fragmentação dos serviços de saúde desempenharam contribuições importantes na dinâmica da doença.

Os achados dialogam com evidências de outros contextos nacionais e internacionais. Enquanto estudos em cidades como o Rio de Janeiro^21^e em países de alta carga de tuberculose^2,22^reportaram queda abrupta nas notificações e dificuldades na retomada dos indicadores pós-pandemia, esses achados evidenciam que, em São Paulo, a subnotificação e a piora dos desfechos persistem nesse período, se distribuindo de forma desigual no estado.

Em países como Austrália e Singapura, os impactos foram menos intensos em comparação com outros países da Ásia e das Américas. Um estudo multinacional apontou que a implementação de estratégias de vigilância e o uso de tecnologias digitais em saúde podem ter contribuído para mitigar os efeitos negativos da pandemia nesses contextos^23^.

O recrudescimento da tuberculose também foi observado em outros países, sendo que na França houve a redução na detecção de casos no início de 2020, seguida por aumento da mortalidade no mesmo período, comportamento distinto do observado no estudo, em que o aumento da mortalidade ocorreu a partir de 2021^24^.

A aplicação do método de decomposição STL identificou mudanças nas tendências das taxas de incidência e mortalidade, bem como nos indicadores operacionais de cura, óbito e interrupção de tratamento, ao longo dos diferentes períodos analisados.

A escolha de marcos temporais baseados na cobertura vacinal contra covid-19 e no encerramento da emergência sanitária nacional possibilitou uma análise mais precisa dos efeitos diretos e indiretos da pandemia sobre a dinâmica da tuberculose, em consonância com recomendações internacionais para estudos de séries temporais em saúde pública^25^.

Em relação à incidência, embora tenha sido reduzida no início da pandemia, o estudo revela forte evidência de subnotificação, principalmente no período da pandemia, o que comprometeu a capacidade dos serviços de identificar e notificar adequadamente os casos, conforme evidenciado em outros contextos^26^.

A falta de diagnóstico e tratamento oportuno podem justificar em parte a tendência crescente de casos, e, portanto, recrudescimento, observada a partir do segundo semestre de 2021, atingindo níveis historicamente elevados até o final de 2023, especialmente em grandes centros urbanos e regiões metropolitanas.

O padrão sugere a ocorrência de um acúmulo de pessoas não diagnosticadas no período pandêmico e posterior recrudescimento da transmissão. Isso pode ser um dos fatores ligados ao aumento de mortalidade por tuberculose no estado de São Paulo a partir do final de 2020, uma vez que o acesso e acompanhamento do tratamento também apresentaram quedas abruptas e tendências de redução após a pandemia.

Os achados acenam para a subnotificação e piora nos desfechos, associadas ao aumento consistente de fatores de risco sociais, como etilismo, tabagismo e uso de drogas, no período pós-pandêmico^7^, reforçando assim a necessidade de políticas integradas que considerem os determinantes sociais da saúde.

Na análise do recrudescimento da tuberculose, observou-se aumento significativo no consumo de drogas (22,4%), álcool (22,1%) e tabaco (29,3%) no período pós-pandêmico, possivelmente relacionado ao estresse e às mudanças nas condições de vida durante a pandemia de covid-19.

Esse aumento pode estar associado à interrupção do tratamento e à recidiva da doença^27,28^. Tal situação reforça a necessidade de ações intersetoriais e abordagens sistêmicas e integradas, da saúde, assistência social e demais setores, com possibilidades de inclusão e desenvolvimento de programas de redução de danos e suporte à cessação do uso de substâncias^28,29^.

Outro resultado importante foi a redução de detecção de casos (de 14,4% no pré-pandemia para 8,2% no pós-pandemia), entre as populações privadas de liberdade no pós-pandemia, o que possivelmente está relacionado à subnotificação. É possível que essa queda esteja ligada à falta de políticas e priorização das populações^8,29^.

A partir das análises espaciais, observou-se formação e intensificação de *clusters*, quando considerada a incidência. Essa conformação pode estar associada, além das questões da detecção, à piora nas condições de vida da população, como alta densidade populacional, condições insalubres e fragilidades na qualidade dos serviços adscritos a essas localidades, notadamente da atenção primária à saúde^30,31^.

Na região de Presidente Prudente, observou-se a formação de *clusters* de alta incidência, com a presença de grandes complexos prisionais^32^ e baixa densidade populacional^33^, o que sugere que a vigilância e o cuidado em saúde nesses ambientes ainda são insuficientes para conter a transmissão.

Em relação aos aglomerados de mortalidade por tuberculose, observou-se a formação de *clusters*, principalmente em regiões com alta densidade populacional e baixa cobertura da atenção primária à saúde, como a Baixada Santista, Grande São Paulo e região de Taubaté^33^. Essas áreas concentram importantes polos econômicos com intensa circulação de pessoas, mas também apresentam elevados níveis de desigualdade social.

Destaca-se que a Região Metropolitana de São Paulo, em 2021, registrava mais de 1 milhão de famílias em situação de pobreza – correspondendo a 54% de todas as famílias nessa condição no estado^34^. A discrepância observada entre o perfil demográfico esperado e os dados da casuística, com predomínio de casos entre pessoas de raça/cor branca, pode estar relacionada à distribuição populacional do estado e à subnotificação em grupos historicamente mais vulneráveis, agravada pela ausência de dados completos sobre raça/cor e escolaridade no período pós-pandêmico. A alta proporção de dados ausentes limita análises mais detalhadas sobre equidade e demanda aprimoramento dos sistemas de informação^3,7^.

Entre as limitações do estudo, destaca-se o uso de dados secundários, que podem ocultar heterogeneidades intrarregionais e mascarar potenciais áreas silenciosas, visto que o estudo se utiliza de dados registrados pelos serviços de saúde e refletem apenas casos conhecidos.

Além disso, a ausência de informações completas sobre variáveis sociodemográficas e clínicas em parte dos registros compromete a precisão das análises, especialmente no que diz respeito ao perfil dos casos e aos desfechos de tratamento. Recomenda-se cautela na generalização dos resultados e a realização de estudos qualitativos para aprofundar a compreensão dos determinantes locais dos *clusters* identificados no estudo.

## CONCLUSÃO

A análise do recrudescimento da tuberculose no estado de São Paulo pós-pandemia de covid-19 evidenciou um agravamento significativo do cenário epidemiológico. Observou-se redução de 1,03% ao mês nas taxas de cura em municípios de grande porte e aumento expressivo nas interrupções de tratamento, que atingiram 2,78% de aumento ao mês em municípios de médio porte. Paralelamente, a mortalidade por tuberculose apresentou uma tendência crescente no período pós-pandêmico, refletindo impactos desiguais nos diferentes territórios.

Os achados evidenciam *clusters* persistentes, e ainda surgimento de novos epicentros da tuberculose no estado, refletindo um cenário global documentado pela Organização Mundial da Saúde. Isso reforça a urgência de realinhamento com a estratégia “*End* TB*”*, cujas ações essenciais incluem investimento em pesquisa e inovação, tratamento preventivo, diagnóstico precoce (preferencialmente de base molecular), tratamento oportuno, estratégias de monitoramento e adesão, e sistemas de proteção social. Acresce-se ainda o preparo para futuras emergências, com sistemas de vigilância resilientes, capazes de manter serviços essenciais de tuberculose durante crises.

## Data Availability

Todos os dados que sustentam os achados do estudo estão contidos no manuscrito. Informações detalhadas adicionais e dados brutos serão disponibilizados mediante solicitação ao autor correspondente.
